# A comprehensive study for selecting optimal treatment modalities for blood cancer in a Fermatean fuzzy dynamic environment

**DOI:** 10.1038/s41598-024-51942-7

**Published:** 2024-01-22

**Authors:** Dilshad Alghazzawi, Aqsa Noor, Hanan Alolaiyan, Hamiden Abd El-Wahed Khalifa, Alhanouf Alburaikan, Songsong Dai, Abdul Razaq

**Affiliations:** 1 Department of Mathematics, College of Science and Arts, King Abdul Aziz University, Rabigh, Saudi Arabia; 2 Division of Science and Technology, Department of Mathematics, University of Education, Lahore, 54770 Pakistan; 3 Department of Mathematics, King Saud University, Riyadh, Saudi Arabia; 4 Department of Mathematics, College of Science and Arts, Qassim University, 51951 Al-Badaya, Saudi Arabia; 5 Department of Operations and Management Research, Faculty of Graduate Studies for Statistical Research, Cairo University, Giza, 12613 Egypt; 6 School of Electronics and Information Engineering, Taizhou University, Taizhou, Zhejiang China

**Keywords:** Applied mathematics, Pure mathematics

## Abstract

Cancer is characterized by uncontrolled cell proliferation, leading to cellular damage or death. Acute lymphoblastic leukemia (ALL), a kind of blood cancer, that affects lymphoid cells and is a challenging malignancy to treat. The Fermatean fuzzy set (FFS) theory is highly effective at capturing imprecision due to its capacity to incorporate extensive problem descriptions that are unclear and periodic. Within the framework of this study, two innovative aggregation operators: The Fermatean fuzzy Dynamic Weighted Averaging (FFDWA) operator and the Fermatean fuzzy Dynamic Weighted Geometric (FFDWG) operator are presented. The important attributes of these operators, providing a comprehensive elucidation of their significant special cases has been discussed in details. Moreover, these operators are utilized in the development of a systematic approach for addressing scenarios involving multiple attribute decision-making (MADM) problems with Fermatean fuzzy (FF) data. A numerical example concerning on finding the optimal treatment approach for ALL using the proposed operators, is provided. At the end, the validity and merits of the new method to illustrate by comparing it with the existing methods.

## Introduction

An essential component of the decision sciences, multi-attribute decision making (MADM) is a procedure that ranks finite alternatives based on the attribute values. The issue of MADM has become intricately linked to the development of businesses and social decision-making in recent years, resulting in its extensive application across various domains. Efficiently and precisely expressing the attribute value emerges as a critical issue in practical decision-making processes. In practice, the utilization of exact values to represent attribute values of alternatives is insufficient due to the intricacy of decision-making problems and the ambiguity of decision-making environments.

In 1965, Zadeh first presented the theory of fuzzy sets (FS)^1^. Its notable accomplishments can be attributed to its adeptness in managing uncertainty. In the academic literature, numerous higher order fuzzy sets have been introduced in recent decades. Atanassov proposed the notion of intuitionistic fuzzy sets (IFS) in 1986^2^. A fuzzy set is defined solely by its membership function, while IFS is distinguished by three parameters: the membership function, the nonmembership function, and the hesitation margins. One of the primary benefits of the IFS is its ability to accommodate uncertainty that may arise from information impression. Consequently, IFS is applicable to a vast array of disciplines, particularly decision making.

Yager extended the IFS concept by defining the Pythagorean fuzzy set (PFS)^3^ in 2013. In PFS, the total of the squares of the degrees of membership and non-membership falls within the interval $\left[\mathrm{0,1}\right]$. In this instance, PFSs can handle more ambiguous situations than IFSs. Consequently, PFS is more effective than IFS at solving real-world problems. In a short period of time, PFSs have piqued the interest of numerous researchers. Using Pythagorean fuzzy aggregation operators, Yager^4^ devised a useful decision-making method to resolve multi criteria decision-making (MCDM) problems. Yager and Abbasov^5^ elucidated the relationship between complex numbers and Pythagorean membership grades, as well as the concepts associated with PFSs. Reformat and Yager^6^ utilized PFNs in 2014 to manage the collaborative-based recommender system. In 2016, Gou et al.^7^ devised a few types of Pythagorean fuzzy mappings and exhaustively analyzed their fundamental properties, including differentiability, continuity, and derivability. In 2018, Zeng et al.^8^ devised an aggregation strategy for the PFS to address MADM issues. Zhang^9^ presented the MCDM technique in 2016 in light of the similarity measure concept. Moreover, PFSs have been successfully linked to numerous domains, including investment decisions^10,11^, candidate selection for the Asian Infrastructure Investment Bank^12^, and domestic airline service quality^13^.

Although the PFS framework is applicable to a broader range of situations, there are still certain situations where PFS cannot be applied. For example, if a person has to rate the degree to which an alternative ${\Xi }_{i}$, with membership and non-membership values of 0.9 and 0.6, respectively, meets or fails a specified criterion ${\varnothing }_{j}$. Here, PFS fails to tackle this situation because ${0.9}^{2}+{0.6}^{2}>1$. Senapati and Yager^14^ introduced the concept of Fermatean fuzzy sets (FFS) to generalize both IFS and PFS. In 2007, IF aggregation operators were introduced by Xu^15^. Xu and Yager^16^ conducted an exhaustive examination of geometric operators within the IF framework. Generalized ordered weighted averaging operators for interval-valued data were thoroughly examined by Li^17^. Induced geometric aggregation operations were introduced to IFS by Wei^18^. In PF frameworks, geometric and arithmetic operators have been examined by Yager^4^. Power aggregation operators were implemented by Wei and Lu in PF- knowledge decision-making^19^. Yager^20^ resolved a multitude of MCDM challenges by employing an assortment of aggregation operators. Novel logarithmic function operational principles were proposed by Garg^21^, resulting in the development of geometric operators and PFS-specific weighted averaging. Garg^22^ suggested PF weighted average and geometric operators to resolve MADM issues. Furthermore, symmetric PF weighted geometric and averaging operators were proposed by Ma and Xu^23^. Subtraction, division, and arithmetic mean operations were introduced to FFSs by in reference^24^. The concept of FF weighted averaging and geometric operators was introduced in^25^. Mishra and Rani^26^ suggested FF weighted aggregated sum product evaluation. Garg et al.^27^ demonstrated working FF aggregation in the COVID-19 testing facility. Yang et al.^28^ investigated the derivatives and continuities of FF functions. Sergi and Sari^29^ put forth a variety of approaches for FF capital budgeting. Sahoo^30^ investigated the applicability of a variety of FFS scoring functions to transportation decision-making. The extensive range of study undertaken on FFSs demonstrates the profound interest of experts in this subject^31–38^.

Cancer is a major cause of death, characterized by the uncontrolled proliferation of aberrant cells in a specific area of the body, leading to cellular dysfunction^39^. It has a significant impact on global mortality rates^40^, with cancer responsible for a worldwide death toll of 10 million individuals in 2022. Blood cancer, a form of neoplastic disorders, manifests within the hematopoietic system and affects blood cells. The etiology of blood cancer primarily stems from genetic mutations or alterations occurring within the DNA of hematopoietic cells^41^.

Leukemia, a form of hematologic malignancy, is distinguished by the unrestrained proliferation of malignant leukocytes within the bone marrow, the principal site of hematopoiesis in the human organism^42^. Clinical manifestations include hemorrhagic tendencies, musculoskeletal discomfort, asthenia, pyrexia, and heightened susceptibility to infectious pathogens due to the deficiency of normal blood cells. The actual cause of leukemia remains unknown, but it is thought to be a combination of genetic and non-inherited environmental elements. Risk factors for developing certain conditions include smoking, exposure to ionizing radiation, exposure to petrochemicals, previous chemotherapy treatment, and the presence of Down syndrome^43^.

Leukemia has four main types: acute lymphoblastic leukemia (ALL), chronic lymphocytic leukemia (CLL), chronic myeloid leukemia (CML), and several rarer variants. Both children and adults develop acute lymphoblastic leukemia (ALL), a progressive cancer. ALL treatment includes remission induction, consolidation, and long-term maintenance^44^.

Hematopoietic stem cell transplantation (HSCT) is one of the most innovative medical operations, however it can cause problems including graft-versus-host disease^45^. Researchers have endeavored to develop innovative and effective therapies that are devoid of these aforementioned complications. In the consolidation phase, giving children with Philadelphia chromosome-positive ALL targeted drugs like Imatinib mesylate along with standard chemotherapy has shown to improve survival rates^46,47^.

In the last decade, the use of immunotherapies based on the body's own T cells has become a new way to treat ALL, aiming to prevent the blood cancer from becoming resistant to chemotherapy.

### Motivation

Dynamic aggregation operators are employed to handle uncertainties and imprecise information that evolves over time. These operators facilitate the collection of data from different time periods to generate an accurate representation, enabling a holistic understanding of the problems. Thus, it becomes important to conduct research that focuses on addressing the issues related to dynamic fuzzy MADM.

### Research gap and objectives of the study

Prior research has concentrated primarily on circumstances involving decision-making in which all initial decision information is gathered simultaneously. Nevertheless, it is customary in a great number of contexts involving decision-making to gather the essential data pertaining to the decision at various time intervals. Dynamic intuitionistic fuzzy MADM was investigated by Xu and Yager^48^, who also suggested the development of dynamic intuitionistic fuzzy aggregation operators. The "time degree" concept is employed in dynamic aggregation operators that gauge a decision maker's information preference across time intervals using a time degree function within the MADM context^49–52^. Introducing the variable in the dynamic framework allows for tracking the temporal evolution of membership degrees and analyzing variations within set time intervals. This capability enhances decision precision, provides insights into changes, and evaluates fuzzy set dynamics. To tackle these challenges, it is critical to formulate additional approaches.

Fuzzy dynamic weighted averaging and geometric operators play a crucial role in improving decision-making models by accommodating dynamic changes and capturing complex relationships in systems affected by uncertainty and imprecision. While these operators are defined for classical fuzzy, intuitionistic fuzzy, and Pythagorean fuzzy environments, their discussion in the literature regarding data containing Fermatean fuzzy sets is lacking. To address this gap, it is essential to define these operators for Fermatean fuzzy sets to handle such situations.

**Figure 1:**
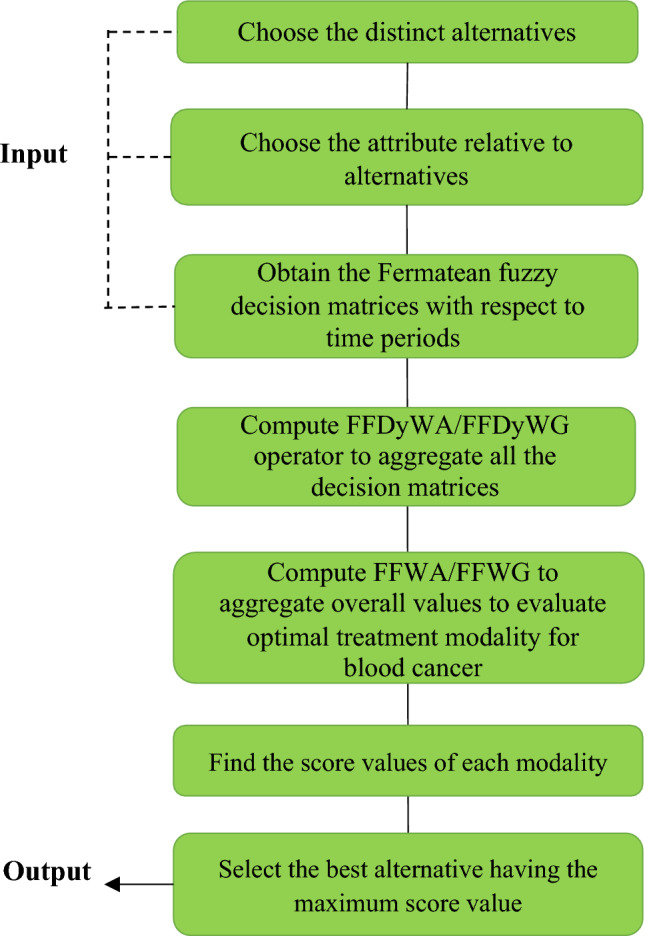
Flowchart of the proposed scheme.

The following constitutes a summary of the principal contributions to this work:

i. Two novel aggregation operators, FFDyWA and FFDyWG, have been developed to handle intricate decision-making situations that incorporate Fermatean fuzzy data.
ii. The basic features of the FFDyWG and FFDyWA operators, encompassing monotonicity, idempotency, and boundedness, have been thoroughly examined.
iii. A systematic approach to resolving MADM problems is provided through the utilization of newly defined operators. The flowchart of the proposed approach is presented is Fig. 1.
iv. The suggested methodology is implemented to tackle a specific MADM challenge that revolves around ascertaining the most effective treatment approach for ALL. It shows the importance of FFDyWA and FFDyWG in the decision-making process.
v. The efficacy of the proposed methodologies is evaluated through an exhaustive comparative analysis with a range of established techniques. The comparison results demonstrate that the devised methodology is dependable and consistent.

The remaining part of this paper is organized as follows: "Preliminaries" section provides the necessary background definitions to understand the key findings of this paper. "Dynamic operations on Fermatean fuzzy numbers" section introduces dynamic aggregate operators for FFS and analyzes their inherent characteristics. In "Application of proposed FF dynamic weighted aggregation operators in MADM problem" section, we introduce a novel approach to address MADM problems using FF information through the application of FF Dynamic weighted aggregation operators. "An optimal treatment modality to cure blood cancer under FF dynamic environment" section is dedicated to illustrating the application of the proposed approach for selecting the optimal treatment modality for curing blood cancer. Furthermore, a comparative analysis is conducted to assess the effectiveness and feasibility of this innovative methodology in contrast to conventional approaches. "Conclusions" section summarizes the conclusion of this entire study.

## Preliminaries

In this section, we provide necessary background definitions to understand the key findings of this paper.

### Definition 1

(^2^). Assume that $X$ is a universe of discourse. The IFS $F$ of $X$ is formally delineated as follows: $F=\{ \langle x, {\mu }_{F}\left(x\right), {\nu }_{F}\left(x\right)\rangle :x\in X\}$, where, ${\mu }_{F} :X \to [\mathrm{0,1}]$ and ${\nu }_{F} :X \to [\mathrm{0,1}]$, are membership functions. The former represents the membership function, while the latter represents the non-membership function. These membership functions must satisfy the constraint $0\le {\mu }_{F}\left(x\right)+ {\nu }_{F}\left(x\right)\le 1$, for all $x\in X$.

IFSs fail to handle the situation when the combined sum of membership and non-membership values exceeds, for this reason^3^ developed the concept of PFSs. This concept is reviewed in the following definition:

### Definition 2

(^3^). The PFS $F$ of the universe $X$ is formally characterized as follows: $F=\{ \langle x, {\mu }_{F}\left(x\right), {\nu }_{F}\left(x\right)\rangle :x\in X\}$, wherein, ${\mu }_{F} :X \to [\mathrm{0,1}]$ and ${\nu }_{F} :X \to [\mathrm{0,1}]$, are membership functions. The former represents the membership function, while the latter represents the non-membership function. These membership functions must satisfy the constraint $0\le {\mu }_{F}^{2}(x)+ {\nu }_{F}^{2}(x)\le 1$, for all $x\in X$.

A more advanced version of FSs was required to account for circumstances that exceeded the scope of the IFS and PFS. The authors introduced the concept of FFSs in^14^ for this purpose; it is defined in the following section.

### Definition 3

(^14^). An FFS $F$ of $X$, is an object of the form: $F=\{ \langle x, {\mu }_{F}\left(x\right), {\nu }_{F}\left(x\right)\rangle :x\in X\}$, where, ${\mu }_{F} :X\to [\mathrm{0,1}]$ and ${\nu }_{F} :X\to [\mathrm{0,1}]$, respectively represents the membership and non-membership functions, satisfying $0\le {\mu }_{F}^{3}(x)+{\nu }_{F}^{3}(x)\le 1$, $\forall x\in X$. The pairs ${\mu }_{F}\left(x\right)$ and ${\nu }_{F}\left(x\right)$ represent, membership and non-membership degrees of the element $:x\in X$. Moreover, for any FFS $F$ and any element $x\in X$, the indeterminacy degree of $x$ with respect to $F$, denoted as ${\pi }_{F}\left(x\right)= \sqrt[3]{1-{\mu }_{F}^{3}\left(x\right)-{\nu }_{F}^{3}(x)}$. Furthermore, we represent the membership and non-membership degrees of an element $x\in X$ as $x=({\mu }_{F},{\nu }_{F})$, defining it as a Fermatean fuzzy number (FFN), where , ${\mu }_{F}\in [\mathrm{0,1}]$, ${\nu }_{F}\in [\mathrm{0,1}]$ and $0\le {\mu }_{F}^{3}+{\nu }_{F}^{3}\le 1$.

### Definition 4

(^14^). For a FFN $F$ that is characterized by its membership function ${\mu }_{F}$ and non-membership function ${\nu }_{F}$, two essential functions are defined as follows:

i. The score function, denoted as $g(F)$, is formulated as $g\left(F\right)={\mu }_{F}^{3}- {\nu }_{F}^{3}$, where the result lies within the closed interval $[-1, 1].$
ii. The accuracy function, denoted as $H(F)$, is expressed as $H\left(F\right)={\mu }_{F}^{3}+ {\nu }_{F}^{3}$, with the outcome residing within the interval $[0, 1].$

Moreover, any two FFNs ${F}_{1}$ and ${F}_{2}$ satisfy the following comparison laws:

i. If $$g\left({F}_{1}\right)>g\left({F}_{2}\right)$$, then $${F}_{1}>{F}_{2}$$
ii. If $g\left({F}_{1}\right)<g\left({F}_{2}\right)$, then ${F}_{1}<{F}_{2}$
iii. If $g\left({F}_{1}\right)=g\left({F}_{2}\right)$, then $H\left({F}_{1}\right)>H\left({F}_{2}\right)$ $\Rightarrow {F}_{1}>{F}_{2}$, $H\left({F}_{1}\right)<H\left({F}_{2}\right)$ ${\Rightarrow F}_{1}<{F}_{2}$ and $H\left({F}_{1}\right)=H\left({F}_{2}\right)$ $\Rightarrow {F}_{1}\sim {F}_{2}$

### Definition 5

(^25^). Consider a collection $\varphi$ having $n$ number of FFNs denoted as ${F}_{i}=({\mu }_{{F}_{i}}, {\nu }_{{F}_{i}})$, where $i = 1, 2, ..., n$, and let $\omega ={\left[{\omega }_{1}, {\omega }_{2},...,{\omega }_{n}\right]}^{T}$ represent the weight vector associated with these FFNs, satisfying the constraints ${\omega }_{i}\in \left[\mathrm{0,1}\right]$ and ${\sum }_{i=1}^{n}{\omega }_{i}=1$. In this context, we describe the concept of a Fermatean fuzzy weighted average (FFWA) operator as a mapping $FFWA:{\varphi }^{n}\to \varphi$, defined by the rule:

$$FFWA\left({F}_{1}{,F}_{2}, ...,{F}_{n}\right)=\left(\sum_{i=1}^{n}{\omega }_{i}{\mu }_{{F}_{i}},\sum_{i=1}^{n}{\omega }_{i}{\nu }_{{F}_{i}}\right)$$

### Definition 6

(^25^). Let ${F}_{i}=({\mu }_{{F}_{i}}, {\nu }_{{F}_{i}})$, where $i = 1, 2, ..., n,$ represent a collection $\varphi$ having $n$ number of FFNs, and let $FFWG:{\varphi }^{n}\to \varphi$ be an operator defined to process these FFNs. The FFWG operator, infact known as the Fermatean fuzzy weighted geometric operator, is defined as follows:

$$FFWG\left({F}_{1}{,F}_{2}, ...,{F}_{n}\right)=\left({\prod }_{i=1}^{n}{\mu }_{{F}_{i}}^{{\omega }_{i}} , {\prod }_{i=1}^{n}{\nu }_{{F}_{i}}^{{\omega }_{i}}\right)$$

In this context, $\omega ={\left[{\omega }_{1}, {\omega }_{2},...,{\omega }_{n}\right]}^{T}$ represents the weight vector associated with the FFNs ${F}_{i}$, with the stipulations that ${\omega }_{i}\in \left[\mathrm{0,1}\right]$ and ${\sum }_{i=1}^{n}{\omega }_{i}=1$.

### Definition 7

(^48^). In the context of a time variable designated $t$, we call ${F}_{t}=({\mu }_{t}, {\nu }_{t})$ as an IF variable, where ${\mu }_{t}\in \left[\mathrm{0,1}\right],{\nu }_{t}\in \left[\mathrm{0,1}\right]$, and ${\mu }_{t}+{\nu }_{t}\le 1$. For an IF variable ${F}_{t}$, if we have a time sequence $t=({t}_{1}, {t}_{2},...,{t}_{p})$, then ${F}_{{t}_{1}},{F}_{{t}_{2}},...,{F}_{{t}_{p}}$ represents $p$ IFNs collected at $p$ different time points.

## Dynamic operations on Fermatean fuzzy numbers

In this section, we explore dynamic operations within the context of the FF environment. It is imperative to emphasize that the applicability of the FFW aggregation operators is restricted to scenarios where FF information possesses time-independent attributes. In cases where time considerations are relevant, as in the collection of FF data across distinct time intervals, the FFW aggregation operators are unsuitable for effectively handling such situations.

### Dynamic operational laws of FFNs

#### Definition 8

Let's consider a time variable, denoted as $t.$ We refer to ${F}_{t}=({\mu }_{t}, {\nu }_{t})$ as a FF variable, where ${\mu }_{t}\in \left[\mathrm{0,1}\right],{\nu }_{t}\in \left[\mathrm{0,1}\right]$, and ${\mu }_{t}^{3}+{\nu }_{t}^{3}\le 1$.

For a FF variable ${F}_{t}$, if we have a time sequence $t=({t}_{1}, {t}_{2},...,{t}_{p})$, then ${F}_{{t}_{1}},{F}_{{t}_{2}},...,{F}_{{t}_{p}}$ represents $p$ FFNs collected at $p$ different time points.

In the following definition, we introduce dynamic operational laws applied to FFNs.

#### Definition 9

Consider two FFNs ${F}_{{t}_{1}}=\left({\mu }_{{t}_{1}},{\nu }_{{t}_{1}}\right)$ and ${F}_{{t}_{2}}=\left({\mu }_{{t}_{2}},{\nu }_{{t}_{2}}\right)$. The following are the fundamental operational laws that regulate their intertheme:

i. $${F}_{{t}_{1}}\le {F}_{{t}_{2}}$$, if $${\mu }_{{t}_{1}}\le {\mu }_{{t}_{2}}$$ and $${\nu }_{{t}_{1}}\le {\nu }_{{t}_{2}}$$
ii. ${F}_{{t}_{1}}={F}_{{t}_{2}}$ if and only if ${F}_{{t}_{1}}\subseteq {F}_{{t}_{2}}$ and ${F}_{{t}_{2}}\subseteq {F}_{{t}_{1}}$
iii. $${F}_{{t}_{1}}^{c}=\left({\nu }_{{t}_{1}},{\mu }_{{t}_{1}}\right)$$

#### Definition 10

Consider ${F}_{t}=\left({\mu }_{t},{\nu }_{t}\right)$, ${F}_{{t}_{1}}=\left({\mu }_{{t}_{1}},{\nu }_{{t}_{1}}\right)$ and ${F}_{{t}_{2}}=\left({\mu }_{{t}_{2}},{\nu }_{{t}_{2}}\right)$ be three FFNs and ${\epsilon }_{t}>0$. These FFNs are subjected to dynamic operations as delineated below:

- $${F}_{{t}_{1}}\oplus {F}_{{t}_{2}}=\left(\sqrt[3]{{\mu }_{{t}_{1}}^{3}+{\mu }_{{t}_{2}}^{3}-{\mu }_{{t}_{1}}^{3}{\mu }_{{t}_{2}}^{3}}, {\nu }_{{t}_{1}}{\nu }_{{t}_{2}}\right)$$
- $${F}_{{t}_{1}}\otimes {F}_{{t}_{2}}=\left({\mu }_{{t}_{1}}{\mu }_{{t}_{2}},\sqrt[3]{{\nu }_{{t}_{1}}^{3}+{\nu }_{{t}_{2}}^{3}-{\nu }_{{t}_{1}}^{3}{\nu }_{{t}_{2}}^{3}}\right)$$
- $${\epsilon }_{t}{F}_{t}=\left(\sqrt[3]{1-{\left(1-{\mu }_{t}^{3}\right)}^{{\epsilon }_{t}}} , {\nu }_{t}^{{\epsilon }_{t}}\right)$$
- $${F}_{t}^{{\epsilon }_{t}}=\left({\mu }_{t}^{{\epsilon }_{t}}, \sqrt[3]{1-{\left(1-{\nu }_{t}^{3}\right)}^{{\epsilon }_{t}}}\right)$$

### Structural properties of FFDyWA operator

Here, we introduce the notion of FFDyWA operator and prove its basic structural attributes.

#### Definition 11

Consider a collection $\varphi$ having $p$ number of FFNs ${F}_{{t}_{k}}=({\mu }_{{t}_{k}},{\nu }_{{t}_{k}})$ at different time periods ${t}_{k}$, where $k=\mathrm{1,2}, \dots , p$. A Fermatean fuzzy dynamic weighted averaging operator is a mapping $FFDyWA:{\varphi }^{p}\to \varphi$, defined as follows:

$$\begin{aligned} & FFDyWA\left( {F_{{t_{1} }} ,F_{{t_{2} }} ,...,F_{{t_{p} }} } \right) = \oplus_{k = 1}^{p} \epsilon_{{t_{k} }} F_{{t_{k} }} \\ & \quad = \left( {\sqrt[3]{{1 - \mathop \prod \limits_{k = 1}^{p} \left( {1 - \mu_{{t_{k} }}^{3} } \right)^{{\epsilon_{{t_{k} }} }} }}, \mathop \prod \limits_{k = 1}^{p} \nu_{{t_{k} }}^{{\epsilon_{{t_{k} }} }} } \right) \\ \end{aligned}$$

Here ${\epsilon }_{t}={[{\epsilon }_{{t}_{1}},{\epsilon }_{{t}_{2}},...,{\epsilon }_{{t}_{p}}] }^{T}$ represents the associated weight vector for the time periods ${t}_{k}$, where $k=\mathrm{1,2},\dots ,p$, such that ${\epsilon }_{{t}_{k}}\in \left[\mathrm{0,1}\right]$ and ${\sum }_{k=1}^{p}{\epsilon }_{{t}_{k}}=1$.

Definition 11 is elaborated upon in the subsequent examples.

#### Example 1

Consider three FFNs ${F}_{{t}_{1}}=\left(\mathrm{0.7,0.6}\right)$, ${F}_{{t}_{2}}=\left(\mathrm{0.8,0.7}\right)$ and ${F}_{{t}_{3}}=\left(\mathrm{0.9,0.5}\right)$. We have ${{\epsilon }_{t}=\left[\mathrm{0.25,0.3,0.45 }\right]}^{T}$ is the associated weight vector assigned to the time periods ${t}_{k},$ where $k=\mathrm{1,2},3$. Then ${\prod }_{k=1}^{3}{\left(1-{\mu }_{{t}_{k}}^{3}\right)}^{{\epsilon }_{{t}_{k}}}=0.403$ and ${\prod }_{k=1}^{3}{\nu }_{{t}_{k}}^{{\epsilon }_{{t}_{k}}}=0.578$. In view of Definition 11, we can obtain:$FFDyWA\left({F}_{{t}_{1}}{,F}_{{t}_{2}}{,F}_{{t}_{3}}\right)= {\oplus }_{k=1}^{3}{\epsilon }_{{t}_{k}}{F}_{{t}_{k}}=\left(0.841, 0.578\right)$.

#### Theorem 1

Consider $p$ numbers of FFNs denoted as ${F}_{{t}_{k}}=({\mu }_{{t}_{k}},{\nu }_{{t}_{k}})$, existing at time intervals ${t}_{k}$, where $k=\mathrm{1,2}, \dots , p$. Let ${\epsilon }_{t}={[{\epsilon }_{{t}_{1}},{\epsilon }_{{t}_{2}},...,{\epsilon }_{{t}_{p}}] }^{T}$ be the weight vector corresponding to ${t}_{k}$, where $k=\mathrm{1,2}, \dots , p$, such that ${\epsilon }_{{t}_{k}}\in \left[\mathrm{0,1}\right]$ and ${\sum }_{k=1}^{p}{\epsilon }_{{t}_{k}}=1$. The aggregated result of these through the FFDyWA operation remains an FFN. It can be expressed as:

$$FFDyWA\left({F}_{{t}_{1}}{,F}_{{t}_{2}},...,{F}_{{t}_{p}}\right)=\left(\sqrt[3]{1-{\prod }_{k=1}^{p}{\left(1-{\mu }_{{t}_{k}}^{3}\right)}^{{\epsilon }_{{t}_{k}}}}, {\prod }_{k=1}^{p}{\nu }_{{t}_{k}}^{{\epsilon }_{{t}_{k}}}\right).$$

#### Proof

The proof of this theorem is established through the utilization of mathematical induction on $p$. We initiate the proof by considering the base case when $p=2$

$$FFDyWA\left({F}_{{t}_{1}}{,F}_{{t}_{2}}\right)={\epsilon }_{{t}_{1}}{F}_{{t}_{1}}\oplus {\epsilon }_{{t}_{2}}{F}_{{t}_{2}}$$

Breaking down the components ${\epsilon }_{{t}_{1}}{F}_{{t}_{1}}$ and ${\epsilon }_{{t}_{2}}{F}_{{t}_{2}}$, in the light of Definition 11 we obtain the following expressions:

$${\epsilon }_{{t}_{1}}{F}_{{t}_{1}}=\left(\sqrt[3]{1-{\left(1-{\mu }_{{t}_{1}}^{3}\right)}^{{\epsilon }_{{t}_{1}}}}, {\nu }_{{t}_{1}}^{{\epsilon }_{{t}_{1}}}\right)$$

$${\epsilon }_{{t}_{2}}{F}_{{t}_{2}}=\left(\sqrt[3]{1-{\left(1-{\mu }_{{t}_{2}}^{3}\right)}^{{\epsilon }_{{t}_{2}}}}, {\nu }_{{t}_{2}}^{{\epsilon }_{{t}_{2}}}\right)$$

Then,

$$\begin{aligned} & \epsilon_{{t_{1} }} F_{{t_{1} }} \oplus \epsilon_{{t_{2} }} F_{{t_{2} }} = \left( {\sqrt[3]{{1 - \left( {1 - \mu_{{t_{1} }}^{3} } \right)^{{\epsilon_{{t_{1} }} }} }}, \nu_{{t_{1} }}^{{\epsilon_{{t_{1} }} }} } \right) \oplus \left( {\sqrt[3]{{1 - \left( {1 - \mu_{{t_{2} }}^{3} } \right)^{{\epsilon_{{t_{2} }} }} }}, \nu_{{t_{2} }}^{{\epsilon_{{t_{2} }} }} } \right) \\ & \quad = \left( {\sqrt[3]{{1 - \left( {1 - \mu_{{t_{1} }}^{3} } \right)^{{\epsilon_{{t_{1} }} }} \left( {1 - \mu_{{t_{2} }}^{3} } \right)^{{\epsilon_{{t_{2} }} }} }}, \nu_{{t_{1} }}^{{\epsilon_{{t_{1} }} }} \nu_{{t_{2} }}^{{\epsilon_{{t_{2} }} }} } \right) \\ \end{aligned}$$

Consequently,

$$FFDyWA\left({F}_{{t}_{1}}{,F}_{{t}_{2}}\right)=\left(\sqrt[3]{1-{\prod }_{k=1}^{2}{\left(1-{\mu }_{{t}_{k}}^{3}\right)}^{{\epsilon }_{{t}_{k}}}}, {\prod }_{k=1}^{2}{\nu }_{{t}_{k}}^{{\epsilon }_{{t}_{k}}}\right)$$

Therefore, the theorem is valid for $p=2$.

Next, we assume that the theorem holds true for $p=n>2$, so we have:

$$\begin{aligned} & FFDyWA\left( {F_{{t_{1} }} ,F_{{t_{2} }} ,...,F_{{t_{n} }} } \right) = \oplus_{k = 1}^{n} \epsilon_{{t_{k} }} F_{{t_{k} }} \\ & \quad = \left( { \sqrt[3]{{1 - \mathop \prod \limits_{k = 1}^{n} \left( {1 - \mu_{{t_{k} }}^{3} } \right)^{{\epsilon_{{t_{k} }} }} }} , \mathop \prod \limits_{k = 1}^{n} \nu_{{t_{k} }}^{{\epsilon_{{t_{k} }} }} } \right) \\ \end{aligned}$$

Now, if $p=n+1$, then

$$\begin{aligned} & FFDyWA\left( {F_{{t_{1} }} ,F_{{t_{2} }} ,...,F_{{t_{n} }} ,F_{{t_{n + 1} }} } \right) = \oplus_{k = 1}^{n} \epsilon_{{t_{k} }} F_{{t_{k} }} \oplus \epsilon_{{t_{n + 1} }} F_{{t_{n + 1} }} \\ & \quad = \left( {\sqrt[3]{{1 - \mathop \prod \limits_{k = 1}^{n} \left( {1 - \mu_{{t_{k} }}^{3} } \right)^{{\epsilon_{{t_{k} }} }} }} , \mathop \prod \limits_{k = 1}^{n} \nu_{{t_{k} }}^{{\epsilon_{{t_{k} }} }} } \right) \oplus \left( {\sqrt[3]{{1 - \left( {1 - \mu_{{t_{n + 1} }}^{3} } \right)^{{\epsilon_{{t_{n + 1} }} }} }} , \nu_{{t_{n + 1} }}^{{\epsilon_{{t_{n + 1} }} }} } \right) \\ \end{aligned}$$

This mean that

$$FFDyWA\left({F}_{{t}_{1}}{,F}_{{t}_{2}},...,{F}_{{t}_{n+1}}\right)=\left( \sqrt[3]{1-{\prod }_{k=1}^{n+1}{\left(1-{\mu }_{{t}_{k}}^{3}\right)}^{{\epsilon }_{{t}_{k}}}}, {\prod }_{k=1}^{n+1}{\nu }_{{t}_{k}}^{{\epsilon }_{{t}_{k}}}\right)$$

This demonstrates that the theorem holds for $p=n+1$. Consequently, we can infer that the statement is valid for all positive integral values of $p$.

The next example provides concrete application of Theorem 1.

#### Example 2

Consider the FFNs ${F}_{{t}_{1}}=\left(\mathrm{0.8,0.7}\right)$, ${F}_{{t}_{2}}=\left(\mathrm{0.6,0.7}\right)$, ${F}_{{t}_{3}}=\left(\mathrm{0.6,0.8}\right)$ and ${F}_{{t}_{4}}=\left(\mathrm{0.9,0.4}\right)$, accompanied by the corresponding weight vectors ${\epsilon }_{t}={[0.2, \mathrm{0.4,0.3,0.1}]}^{T}$ for the time periods ${t}_{k}$, where $k=\mathrm{1,2},\mathrm{3,4}$. Calculations yield: ${\prod }_{k=1}^{4}{\left(1-{\mu }_{{t}_{k}}^{3}\right)}^{{\epsilon }_{{t}_{k}}}=0.641$ and ${\prod }_{k=1}^{4}{\nu }_{{t}_{k}}^{{\epsilon }_{{t}_{k}}}=0.688$. In view of Definition 11, we summarize the above discussion as follows:

$$FFDyWA\left({F}_{{t}_{1}}{,F}_{{t}_{2}}{,F}_{{t}_{3}},{F}_{{t}_{4}}\right)= {\oplus }_{k=1}^{4}{\epsilon }_{{t}_{k}}{F}_{{t}_{k}}=\left(0.710, 0.688\right)$$

Thus, exemplifying the practical application of Theorem 1.

#### Theorem 2

(Idempotency) If all ${F}_{{t}_{k}}=\left({\mu }_{{t}_{k}}, {\nu }_{{t}_{k}}\right)$, where $k=\mathrm{1,2}, \dots , p$ are equal, that is, ${F}_{{t}_{k}}={F}_{{t}_{j}}$ for all $k$ and for some $j\in \{\mathrm{1,2},..., p\}$, where ${F}_{{t}_{j}}=\left({\mu }_{{t}_{j}}, {\nu }_{{t}_{j}}\right)$. Then $FFDWA\left({F}_{{t}_{1}}, {F}_{{t}_{2}},..., {F}_{{t}_{p}}\right)={F}_{{t}_{j}}$.

#### Proof

Given that ${F}_{{t}_{k}}={F}_{{t}_{j}}$ for all $k=\mathrm{1,2},\dots ,p$, and for some $j\in \{\mathrm{1,2},..., p\}$, then ${\mu }_{{t}_{k}}={\mu }_{{t}_{j}}$ and ${\nu }_{{t}_{k}}={\nu }_{{t}_{j}}$.

Consider

$$\begin{aligned} & FFDyWA\left( {F_{{t_{1} }} , F_{{t_{2} }} ,..., F_{{t_{p} }} } \right) = \left( {\sqrt[3]{{1 - \mathop \prod \limits_{k = 1}^{p} \left( {1 - \mu_{{t_{k} }}^{3} } \right)^{{\epsilon_{{t_{k} }} }} }},\mathop \prod \limits_{k = 1}^{p} \nu_{{t_{k} }}^{{\epsilon_{{t_{k} }} }} } \right) \\ & \quad = \left( {\sqrt[3]{{1 - \left( {1 - \mu_{{t_{j} }}^{3} } \right)^{{\mathop \sum \limits_{k = 1}^{p} \epsilon_{{t_{k} }} }} }}, \nu_{{t_{j} }}^{{\mathop \sum \limits_{k = 1}^{p} \epsilon_{{t_{k} }} }} } \right) = \left( {\sqrt[3]{{1 - \left( {1 - \mu_{{t_{j} }}^{3} } \right)}}, \nu_{{t_{j} }} } \right) \\ & \quad= \left( {\sqrt[3]{{\mu_{{t_{j} }}^{3} }}, \nu_{{t_{j} }} } \right) = (\mu_{{t_{j} }} , \nu_{{t_{j} }} ) \\ \end{aligned}$$

Consequently,

$$FFDyWA\left({F}_{{t}_{1}}, {F}_{{t}_{2}},..., {F}_{{t}_{p}}\right)={F}_{{t}_{j}}$$

#### Theorem 3

(Boundedness) Let ${F}_{t}^{-}=\left(\underset{{t}_{k}}{{\text{min}}}\left\{{\mu }_{{t}_{k}}\right\},\underset{{t}_{k}}{{\text{max}}}\left\{{\nu }_{{t}_{k}}\right\}\right)$ and ${F}_{t}^{+}=\left(\underset{{t}_{k}}{{\text{max}}}\left\{{\mu }_{{t}_{k}}\right\},\underset{{t}_{k}}{{\text{min}}}\left\{{\nu }_{{t}_{k}}\right\}\right)$ be the lower and upper bounds of the FFNs ${F}_{{t}_{k}}=\left({\mu }_{{t}_{k}}, {\nu }_{{t}_{k}}\right)$, where $k$ takes on values from $1$ to $p$. Assume that ${\epsilon }_{t}={[{\epsilon }_{{t}_{1}},{\epsilon }_{{t}_{2}},...,{\epsilon }_{{t}_{p}}] }^{T}$ is the corresponding vector of these FFNs, such that ${\epsilon }_{{t}_{k}}\in \left[\mathrm{0,1}\right]$ and ${\sum }_{k=1}^{p}{\epsilon }_{{t}_{k}}=1$. Then.

$${{F}_{t}}^{-}\le FFDyWA\left({F}_{{t}_{1}}, {F}_{{t}_{2}},..., {F}_{{t}_{p}}\right)\le {{F}_{t}}^{+}$$

#### Proof

Consider

$$FFDyWA\left({F}_{{t}_{1}}, {F}_{{t}_{2}},..., {F}_{{t}_{p}}\right)=\left({\mu }_{t}, {\nu }_{t}\right),$$

For each FFN ${\mu }_{{t}_{k}}$, we have

1 $$\begin{gathered} \mathop {\min }\limits_{{t_{k} }} \left\{ {\mu_{{t_{k} }} } \right\} \le \mu_{{t_{k} }} \le \mathop {\max }\limits_{{t_{k} }} \left\{ {\mu_{{t_{k} }} } \right\} \hfill \\ \Rightarrow \mathop {\min }\limits_{{t_{k} }} \left\{ {\mu_{{t_{k} }}^{3} } \right\} \le \mu_{{t_{k} }}^{3} \le \mathop {\max }\limits_{{t_{k} }} \left\{ {\mu_{{t_{k} }}^{3} } \right\} \hfill \\ \Rightarrow 1 - \mathop {\max }\limits_{{t_{k} }} \left\{ {\mu_{{t_{k} }}^{3} } \right\} \le 1 - \mu_{{t_{k} }}^{3} \le 1 - \mathop {\min }\limits_{{t_{k} }} \left\{ {\mu_{{t_{k} }}^{3} } \right\} \hfill \\ \Rightarrow \mathop \prod \limits_{k = 1}^{p} \left( {1 - \mathop {\max }\limits_{{t_{k} }} \left\{ {\mu_{{t_{k} }}^{3} } \right\}} \right)^{{\epsilon_{{t_{k} }} }} \le \mathop \prod \limits_{k = 1}^{p} \left( {1 - \mu_{{t_{k} }}^{3} } \right)^{{\epsilon_{{t_{k} }} }} \le \mathop \prod \limits_{k = 1}^{p} \left( {1 - \mathop {\min }\limits_{{t_{k} }} \left\{ {\mu_{{t_{k} }}^{3} } \right\}} \right)^{{\epsilon_{{t_{k} }} }} \hfill \\ \Rightarrow \left( {1 - \mathop {\max }\limits_{{t_{k} }} \left\{ {\mu_{{t_{k} }}^{3} } \right\}} \right)^{{\mathop \sum \limits_{k = 1}^{p} \epsilon_{{t_{k} }} }} \le \mathop \prod \limits_{k = 1}^{p} \left( {1 - \mu_{{t_{k} }}^{3} } \right)^{{\epsilon_{{t_{k} }} }} \le \left( {1 - \mathop {\min }\limits_{{t_{k} }} \left\{ {\mu_{{t_{k} }}^{3} } \right\}} \right)^{{\mathop \sum \limits_{k = 1}^{p} \epsilon_{{t_{k} }} }} \hfill \\ \Rightarrow \left( {1 - \mathop {\max }\limits_{{t_{k} }} \left\{ {\mu_{{t_{k} }}^{3} } \right\}} \right) \le \mathop \prod \limits_{k = 1}^{p} \left( {1 - \mu_{{t_{k} }}^{3} } \right)^{{\epsilon_{{t_{k} }} }} \le \left( {1 - \mathop {\min }\limits_{{t_{k} }} \left\{ {\mu_{{t_{k} }}^{3} } \right\}} \right) \hfill \\ \Rightarrow \mathop {\min }\limits_{{t_{k} }} \left\{ {\mu_{{t_{k} }}^{3} } \right\} \le 1 - \mathop \prod \limits_{k = 1}^{p} \left( {1 - \mu_{{t_{k} }}^{3} } \right)^{{\epsilon_{{t_{k} }} }} \le \mathop {\max }\limits_{{t_{k} }} \left\{ {\mu_{{t_{k} }}^{3} } \right\} \hfill \\ \Rightarrow \sqrt[3]{{\mathop {\min }\limits_{{t_{k} }} \left\{ {\mu_{{t_{k} }}^{3} } \right\} }} \le \sqrt[3]{{1 - \mathop \prod \limits_{k = 1}^{p} \left( {1 - \mu_{{t_{k} }}^{3} } \right)^{{\epsilon_{{t_{k} }} }} }} \le \sqrt[3]{{\mathop {\max }\limits_{{t_{k} }} \left\{ {\mu_{{t_{k} }}^{3} } \right\}}} \hfill \\ \Rightarrow \mathop {\min }\limits_{{t_{k} }} \left\{ {\mu_{{t_{k} }} } \right\} \le \mu_{t} \le \mathop {\max }\limits_{{t_{k} }} \left\{ {\mu_{{t_{k} }} } \right\} \hfill \\ \end{gathered}$$

Moreover,

2 $$\begin{gathered} \mathop {\min }\limits_{{t_{k} }} \left\{ {\nu_{{t_{k} }} } \right\} \le \nu_{{t_{k} }} \le \mathop {\max }\limits_{{t_{k} }} \left\{ {\nu_{{t_{k} }} } \right\} \hfill \\ \Rightarrow \mathop \prod \limits_{k = 1}^{p} \left( {\mathop {\min }\limits_{{t_{k} }} \left\{ {\nu_{{t_{k} }} } \right\}} \right)^{{\epsilon_{{t_{k} }} }} \le \mathop \prod \limits_{k = 1}^{p} \left( {\nu_{{t_{k} }} } \right)^{{\epsilon_{{t_{k} }} }} \le \mathop \prod \limits_{k = 1}^{p} \left( {\mathop {\max }\limits_{{t_{k} }} \left\{ {\nu_{{t_{k} }} } \right\}} \right)^{{\epsilon_{{t_{k} }} }} \hfill \\ \Rightarrow \left( {\mathop {\min }\limits_{{t_{k} }} \left\{ {\nu_{{t_{k} }} } \right\}} \right)^{{\mathop \sum \limits_{k = 1}^{p} \epsilon_{{t_{k} }} }} \le \mathop \prod \limits_{k = 1}^{p} \left( {\nu_{{t_{k} }} } \right)^{{\epsilon_{{t_{k} }} }} \le \left( {\mathop {\max }\limits_{{t_{k} }} \left\{ {\nu_{{t_{k} }} } \right\}} \right)^{{\mathop \sum \limits_{k = 1}^{p} \epsilon_{{t_{k} }} }} \hfill \\ \Rightarrow \mathop {\min }\limits_{{t_{k} }} \left\{ {\nu_{{t_{k} }} } \right\} \le \nu_{t} \le \mathop {\max }\limits_{{t_{k} }} \left\{ {\nu_{{t_{k} }} } \right\} \hfill \\ \end{gathered}$$

Hence by comparing relation 1 and 2, we obtain that,

$${{F}_{t}}^{-}\le FFDyWA\left({F}_{{t}_{1}}, {F}_{{t}_{2}},..., {F}_{{t}_{p}}\right)\le {{F}_{t}}^{+}.$$

#### Theorem 4

(Monotonicity) Consider two collections $p$ number of FFNs, denoted as ${F}_{{t}_{k}}=\left({\mu }_{{t}_{k}}, {\upnu }_{{{\text{t}}}_{{\text{k}}}}\right)$ and ${G}_{{t}_{k}}=\left({\eta }_{{t}_{k}}, {\xi }_{{t}_{k}}\right)$, with $k$ ranging from $1$ to $p$. Let ${\epsilon }_{t}={[{\epsilon }_{{t}_{1}},{\epsilon }_{{t}_{2}},...,{\epsilon }_{{t}_{p}}] }^{T}$ be the weight vector corresponding to ${t}_{k}$, where $k=\mathrm{1,2}, \dots , p$, such that ${\epsilon }_{{t}_{k}}\in \left[\mathrm{0,1}\right]$ and ${\sum }_{k=1}^{p}{\epsilon }_{{t}_{k}}=1$. If for each ${t}_{k}$, ${\mu }_{{t}_{k}}\le {\eta }_{{t}_{k}}$ and ${\nu }_{{t}_{k}}\ge {\xi }_{{t}_{k}}$, then

$$FFDyWA\left({F}_{{t}_{1}}, {F}_{{t}_{2}},..., {F}_{{t}_{p}}\right)\le FFDyWA\left({G}_{{t}_{1}}, {G}_{{t}_{2}},..., {G}_{{t}_{p}}\right)$$

#### Proof

In view of given presentation of ${F}_{{t}_{k}}$ and ${G}_{{t}_{k}}$ the corresponding FFDyWA’s outcomes as follow:

$$FFDyWA\left({F}_{{t}_{1}}, {F}_{{t}_{2}},..., {F}_{{t}_{p}}\right)=\left({\mu }_{t}, {\nu }_{t}\right)$$

$$FFDyWA\left({G}_{{t}_{1}}, {G}_{{t}_{2}},..., {G}_{{t}_{p}}\right)=\left({\eta }_{t}, {\xi }_{t}\right)$$

Since ${\mu }_{{t}_{k}}\le {\eta }_{{t}_{k}}$, which implies that ${\mu }_{{t}_{k}}^{3}\le {\eta }_{{t}_{k}}^{3}$, we can deduce that

$$\begin{gathered} 1 - \mu_{{t_{k} }}^{3} \ge 1 - \eta_{{t_{k} }}^{3} \hfill \\ \Rightarrow \mathop \prod \limits_{k = 1}^{p} \left( {1 - \mu_{{t_{k} }}^{3} } \right)^{{\epsilon_{{t_{k} }} }} \ge \mathop \prod \limits_{k = 1}^{p} \left( {1 - \eta_{{t_{k} }}^{3} } \right)^{{\epsilon_{{t_{k} }} }} \hfill \\ \Rightarrow 1 - \mathop \prod \limits_{k = 1}^{p} \left( {1 - \mu_{{t_{k} }}^{3} } \right)^{{\epsilon_{{t_{k} }} }} \le 1 - \mathop \prod \limits_{k = 1}^{p} \left( {1 - \eta_{{t_{k} }}^{3} } \right)^{{\epsilon_{{t_{k} }} }} \hfill \\ \Rightarrow \sqrt[3]{{1 - \mathop \prod \limits_{k = 1}^{p} \left( {1 - \mu_{{t_{k} }}^{3} } \right)^{{\epsilon_{{t_{k} }} }} }} \le \sqrt[3]{{1 - \mathop \prod \limits_{k = 1}^{p} \left( {1 - \eta_{{t_{k} }}^{3} } \right)^{{\epsilon_{{t_{k} }} }} }} \hfill \\ \end{gathered}$$

Hence, we can conclude that

3 $${\mu }_{t}\le {\eta }_{t}$$

In a similar way, by considering ${\nu }_{{t}_{k}}\ge {\xi }_{{t}_{k}}$, we derive: ${\prod }_{k=1}^{p}{\nu }_{{t}_{k}}^{{\epsilon }_{{t}_{k}}}\ge {\prod }_{k=1}^{p}{\xi }_{{t}_{k}}^{{\epsilon }_{{t}_{k}}}$ implying that,

4 $${\nu }_{t}\ge {\xi }_{t}$$

Therefore, utilizing Definition 9 and by comparing relation 3 and 4, we obtain that:

$$FFDyWA\left({F}_{{t}_{1}}, {F}_{{t}_{2}},..., {F}_{{t}_{p}}\right)\le FFDyWA\left({G}_{{t}_{1}}, {G}_{{t}_{2}},..., {G}_{{t}_{p}}\right)$$

Thus, the monotonicity property is established.

### Structural properties of $FFDyWG$ operator

This subsection serves to introduce the concept of the FFDyWG operator and outline its fundamental structural characteristics.

#### Definition 12

Consider a collection $\varphi$ having $p$ number of FFNs, denoted as ${F}_{{t}_{k}}=({\mu }_{{t}_{k}},{\nu }_{{t}_{k}})$, existing across distinct time periods ${t}_{k}$, where k takes values from $1$ to $p$. A Fermatean fuzzy dynamic weighted geometric operator is a mapping $FFDyWG$:${\varphi }^{p}\to \varphi$, which is defined as follows:

$$\begin{aligned}&FFDyWG\left({F}_{{t}_{1}}{,F}_{{t}_{2}},...,{F}_{{t}_{p}}\right)={\otimes }_{k=1}^{p}{F}_{{t}_{k}}^{{\epsilon }_{{t}_{k}}}\\&\quad=\left({\prod }_{k=1}^{p}{\mu }_{{t}_{k}}^{{\epsilon }_{{t}_{k}}}, \sqrt[3]{1-{\prod }_{k=1}^{p}{\left(1-{\nu }_{{t}_{k}}^{3}\right)}^{{\epsilon }_{{t}_{k}}}}\right)\end{aligned}$$

Here ${\epsilon }_{t}={[{\epsilon }_{{t}_{1}}, {\epsilon }_{{t}_{2}},..., {\epsilon }_{{t}_{p}}]}^{T}$ represents the weight vector associated with the time periods ${t}_{k}$, where $k=\mathrm{1,2}, \dots , p$, such that ${\epsilon }_{{t}_{k}}\in \left[\mathrm{0,1}\right]$ and ${\sum }_{k=1}^{p}{\epsilon }_{{t}_{k}}=1$.

Following examples elaborate the complete understanding of the above Definition.

#### Example 3

Consider three FFNs ${F}_{{t}_{1}}=\left(\mathrm{0.9,0.3}\right)$, ${F}_{{t}_{2}}=\left(\mathrm{0.8,0.6}\right)$ and ${F}_{{t}_{3}}=\left(\mathrm{0.7,0.8}\right)$ and the ${\epsilon }_{t}={\left[\mathrm{0.25,0.35,0.4 }\right]}^{T}$ is the associated weight vector, assigned to the time periods ${t}_{k},$ where $k=\mathrm{1,2},3$. Then ${\prod }_{k=1}^{3}{\mu }_{{t}_{k}}^{{\epsilon }_{{t}_{k}}}=0.781$ and ${\prod }_{k=1}^{3}{\left(1-{\nu }_{{t}_{k}}^{3}\right)}^{{\epsilon }_{{t}_{k}}}=0.315$. In view of Definition 12, we obtain:

$$FFDyWG\left({F}_{{t}_{1}}{,F}_{{t}_{2}}{,F}_{{t}_{3}}\right)= {\otimes }_{k=1}^{3}{\epsilon }_{{t}_{k}}{F}_{{t}_{k}}=\left(\mathrm{0.781,0.881}\right)$$

#### Theorem 5

Consider $p$ number of FFNs represented as ${F}_{{t}_{k}}=({\mu }_{{t}_{k}},{\nu }_{{t}_{k}})$, corresponding to time periods ${t}_{k},\mathrm{ where} k=\mathrm{1,2}, \dots , p$. Let ${\epsilon }_{t}={[{\epsilon }_{{t}_{1}},{\epsilon }_{{t}_{2}},...,{\epsilon }_{{t}_{p}}] }^{T}$ be the weight vector corresponding to ${t}_{k}$, where $k=\mathrm{1,2}, \dots , p$, such that ${\epsilon }_{{t}_{k}}\in \left[\mathrm{0,1}\right]$ and ${\sum }_{k=1}^{p}{\epsilon }_{{t}_{k}}=1$. Then, the outcome of aggregating these FFNs through the FFDyWG operation yields a FFN as follows:

$$FFDyWG\left({F}_{{t}_{1}}{,F}_{{t}_{2}},...,{F}_{{t}_{p}}\right)=\left({\prod }_{k=1}^{p}{\mu }_{{t}_{k}}^{{\epsilon }_{{t}_{k}}}, \sqrt[3]{1-{\prod }_{k=1}^{p}{\left(1-{\nu }_{{t}_{k}}^{3}\right)}^{{\epsilon }_{{t}_{k}}}}\right)$$

#### Proof

The proof of this theorem is established through the utilization of mathematical induction on $p$. We initiate the proof by considering the base case when $p=2$

$$FFDyWG\left({F}_{{t}_{1}}{,F}_{{t}_{2}}\right)={F}_{{t}_{1}}^{{\epsilon }_{{t}_{1}}}\otimes {F}_{{t}_{2}}^{{\epsilon }_{{t}_{2}}}$$

Breaking down the components ${F}_{{t}_{1}}^{{\epsilon }_{{t}_{1}}}$ and ${F}_{{t}_{2}}^{{\epsilon }_{{t}_{2}}}$, in the light of Definition 12 we obtain the following expressions:

$${F}_{{t}_{1}}^{{\epsilon }_{{t}_{1}}}=\left({\mu }_{{t}_{1}}^{{\epsilon }_{{t}_{1}}},\sqrt[3]{1-{\left(1-{\nu }_{{t}_{1}}^{3}\right)}^{{\epsilon }_{{t}_{1}}}}\right)$$

$${F}_{{t}_{2}}^{{\epsilon }_{{t}_{2}}}=\left({\mu }_{{t}_{2}}^{{\epsilon }_{{t}_{2}}},\sqrt[3]{1-{\left(1-{\nu }_{{t}_{2}}^{3}\right)}^{{\epsilon }_{{t}_{2}}}}\right)$$

Then,

$$\begin{gathered} F_{{t_{1} }}^{{\epsilon_{{t_{1} }} }} \otimes F_{{t_{2} }}^{{\epsilon_{{t_{2} }} }} = \left( {\mu_{{t_{1} }}^{{\epsilon_{{t_{1} }} }} ,\sqrt[3]{{1 - \left( {1 - \nu_{{t_{1} }}^{3} } \right)^{{\epsilon_{{t_{1} }} }} }}} \right) \otimes \left( { \mu_{{t_{2} }}^{{\epsilon_{{t_{2} }} }} ,\sqrt[3]{{1 - \left( {1 - \nu_{{t_{2} }}^{3} } \right)^{{\epsilon_{{t_{2} }} }} }}} \right) \hfill \\ = \left( {\mu_{{t_{1} }}^{{\epsilon_{{t_{1} }} }} \mu_{{t_{2} }}^{{\epsilon_{{t_{2} }} }} ,\sqrt[3]{{1 - (1 - \nu_{{t_{1} }}^{3} )^{{\epsilon_{{t_{1} }} }} (1 - \nu_{{t_{2} }}^{3} )^{{\epsilon_{{t_{2} }} }} }}} \right) \hfill \\ \end{gathered}$$

Consequently,

$$FFDyWG\left({F}_{{t}_{1}}{,F}_{{t}_{2}}\right)=\left({\prod }_{k=1}^{2}{\mu }_{{t}_{k}}^{{\epsilon }_{{t}_{k}}},\sqrt[3]{1-{\prod }_{k=1}^{2}{\left(1-{\nu }_{{t}_{k}}^{3}\right)}^{{\epsilon }_{{t}_{k}}}}\right)$$

Therefore, the theorem is valid for $p=2$.

Next, we assume that the theorem holds true for $p=n>2$, so we have:

$$\begin{gathered} FFDyWG\left( {F_{{t_{1} }} ,F_{{t_{2} }} ,...,F_{{t_{n} }} } \right) = \otimes_{k = 1}^{n} F_{{t_{k} }}^{{\epsilon_{{t_{k} }} }} \hfill \\ = \left( {\mathop \prod \limits_{k = 1}^{n} \mu_{{t_{k} }}^{{\epsilon_{{t_{k} }} }} , \sqrt[3]{{1 - \mathop \prod \limits_{k = 1}^{n} \left( {1 - \nu_{{t_{k} }}^{3} } \right)^{{\epsilon_{{t_{k} }} }} }}} \right) \hfill \\ \end{gathered}$$

Now, if $p=n+1$, then we have

$$\begin{gathered} FFDyWG\left( {F_{{t_{1} }} ,F_{{t_{2} }} ,...,F_{{t_{n} }} ,F_{{t_{n + 1} }} } \right) = \otimes_{k = 1}^{n} F_{{t_{k} }}^{{\epsilon_{{t_{k} }} }} \otimes F_{{t_{n + 1} }}^{{\epsilon_{{t_{n + 1} }} }} \hfill \\ = \left( { \mathop \prod \limits_{k = 1}^{n} \mu_{{t_{k} }}^{{\epsilon_{{t_{k} }} }} , \sqrt[3]{{1 - \mathop \prod \limits_{k = 1}^{n} \left( {1 - \nu_{{t_{k} }}^{3} } \right)^{{\epsilon_{{t_{k} }} }} }}} \right) \otimes \left( { \mu_{{t_{n + 1} }}^{{\epsilon_{{t_{n + 1} }} }} ,\sqrt[3]{{1 - \left( {1 - \nu_{{t_{n + 1} }}^{3} } \right)^{{\epsilon_{{t_{n + 1} }} }} }}} \right) \hfill \\ \end{gathered}$$

This mean that

$$FFDyWG\left({F}_{{t}_{1}}{,F}_{{t}_{2}}, ...,{F}_{{t}_{n+1}}\right)=\left( {\prod }_{k=1}^{n+1}{\mu }_{{t}_{k}}^{{\epsilon }_{{t}_{k}}}, \sqrt[3]{1-{\prod }_{k=1}^{n+1}{\left(1-{\nu }_{{t}_{k}}^{3}\right)}^{{\epsilon }_{{t}_{k}}}}\right).$$

This demonstrates that the theorem holds for $p=n+1$. Consequently, we can infer that the statement is valid for all positive integral values of $p$.

#### Example 4

To exemplify the application of the FFDyWG operator, consider four FFNs ${F}_{{t}_{1}}=\left(\mathrm{0.9,0.4}\right)$, ${F}_{{t}_{2}}=\left(\mathrm{0.8,0.5}\right)$, ${F}_{{t}_{3}}=\left(\mathrm{0.7,0.3}\right)$ and ${F}_{{t}_{4}}=\left(\mathrm{0.6,0.5}\right)$ and the associated weight vector ${\left[\mathrm{0.25,0.47,0.13,0.15}\right]}^{T}$ assigned to the time periods ${t}_{k},$ where $k=\mathrm{1,2},\mathrm{3,4}$. Subsequently, we calculate:

$${\prod }_{k=1}^{4}{\mu }_{{t}_{k}}^{{\epsilon }_{{t}_{k}}}=\left(0.775\right)$$

$${\prod }_{k=1}^{4}{\left(1-{\nu }_{{t}_{k}}^{3}\right)}^{{\epsilon }_{{t}_{k}}}=\left(0.902\right)$$

In view of Definition 12, we summarize the above discussion as follows:

$$FFDyWG\left({F}_{{t}_{1}}{,F}_{{t}_{2}}{,F}_{{t}_{3}},{F}_{{t}_{4}}\right)= {\otimes }_{k=1}^{4}{F}_{{t}_{k}}^{{\epsilon }_{{t}_{k}}}=\left(0.775, 0.461\right).$$

#### Theorem 6

(Idempotency) If all ${F}_{{t}_{k}}=\left({\mu }_{{t}_{k}}, {\nu }_{{t}_{k}}\right)$, where $k=\mathrm{1,2}, \dots , p$ are equal, that is, ${F}_{{t}_{k}}={F}_{{t}_{j}}$ for all $k$ and for some $j\in \{\mathrm{1,2},..., p\}$, where ${F}_{{t}_{j}}=\left({\mu }_{{t}_{j}}, {\nu }_{{t}_{j}}\right)$. Then $FFDyWG\left({F}_{{t}_{1}}, {F}_{{t}_{2}}, ... , {F}_{{t}_{p}}\right)={F}_{{t}_{j}}$.

#### Proof

The proof of this theorem is omitted because it is closely aligning with that of Theorem 2.

#### Theorem 7

(Boundedness) Let ${F}_{{t}_{k}}^{-}=\left(\underset{{t}_{k}}{{\text{min}}}\left\{{\mu }_{{t}_{k}}\right\},\underset{{t}_{k}}{{\text{max}}}\left\{{\nu }_{{t}_{k}}\right\}\right)$ and ${F}_{{t}_{k}}^{+}=\left(\underset{{t}_{k}}{{\text{max}}}\left\{{\mu }_{{t}_{k}}\right\},\underset{{t}_{k}}{{\text{min}}}\left\{{\nu }_{{t}_{k}}\right\}\right)$ be the lower and upper bounds of the FFNs ${F}_{{t}_{k}}=\left({\mu }_{{t}_{k}}, {\nu }_{{t}_{k}}\right)$, where $k$ takes on values from $1$ to $p$. Assume that ${\epsilon }_{t}={[{\epsilon }_{{t}_{1}},{\epsilon }_{{t}_{2}},...,{\epsilon }_{{t}_{p}}] }^{T}$ is the corresponding vector of these FFNs, such that ${\epsilon }_{{t}_{k}}\in \left[\mathrm{0,1}\right]$, and ${\sum }_{k=1}^{p}{\epsilon }_{{t}_{k}}=1$. Then

$${{F}_{t}}^{-}\le FFDyWG\left({F}_{{t}_{1}}, {F}_{{t}_{2}}, ... , {F}_{{t}_{p}}\right)\le {{F}_{t}}^{+}$$

#### Proof

Consider $FFDyWG\left({F}_{{t}_{1}}, {F}_{{t}_{2}},..., {F}_{{t}_{p}}\right)=\left({\mu }_{t}, {\nu }_{t}\right)$. For each ${\mu }_{{t}_{k}}$, we have

5 $$\begin{gathered} \mathop {\min }\limits_{{t_{k} }} \left\{ {\mu_{{t_{k} }} } \right\} \le \mu_{{t_{k} }} \le \mathop {\max }\limits_{{t_{k} }} \left\{ {\mu_{{t_{k} }} } \right\} \hfill \\ \Rightarrow \mathop \prod \limits_{k = 1}^{p} \left( {\mathop {\min }\limits_{{t_{k} }} \left\{ {\mu_{{t_{k} }} } \right\}} \right)^{{\epsilon_{{t_{k} }} }} \le \mathop \prod \limits_{k = 1}^{p} \left( {\mu_{{t_{k} }} } \right)^{{\epsilon_{{t_{k} }} }} \le \mathop \prod \limits_{k = 1}^{p} \left( {\mathop {\max }\limits_{{t_{k} }} \left\{ {\mu_{{t_{k} }} } \right\}} \right)^{{\epsilon_{{t_{k} }} }} \hfill \\ \Rightarrow \left( {\mathop {\min }\limits_{{t_{k} }} \left\{ {\mu_{{t_{k} }} } \right\}} \right)^{{\mathop \sum \limits_{k = 1}^{p} \epsilon_{{t_{k} }} }} \le \mathop \prod \limits_{k = 1}^{p} \left( {\mu_{{t_{k} }} } \right)^{{\epsilon_{{t_{k} }} }} \le \left( {\mathop {\max }\limits_{{t_{k} }} \left\{ {\mu_{{t_{k} }} } \right\}} \right)^{{\mathop \sum \limits_{k = 1}^{p} \epsilon_{{t_{k} }} }} \hfill \\ \Rightarrow \mathop {\min }\limits_{{t_{k} }} \left\{ {\mu_{{t_{k} }} } \right\} \le \mu_{t} \le \mathop {\max }\limits_{{t_{k} }} \left\{ {\mu_{{t_{k} }} } \right\} \hfill \\ \end{gathered}$$

Moreover,

6 $$\begin{gathered} \mathop {\min }\limits_{{t_{k} }} \left\{ {\nu_{{t_{k} }} } \right\} \le \nu_{{t_{k} }} \le \mathop {\max }\limits_{{t_{k} }} \left\{ {\nu_{{t_{k} }} } \right\} \hfill \\ \Rightarrow \mathop {\min }\limits_{{t_{k} }} \left\{ {\nu_{{t_{k} }}^{3} } \right\} \le \nu_{{t_{k} }}^{3} \le \mathop {\max }\limits_{{t_{k} }} \left\{ {\nu_{{t_{k} }}^{3} } \right\} \hfill \\ \Rightarrow 1 - \mathop {\max }\limits_{{t_{k} }} \left\{ {\nu_{{t_{k} }}^{3} } \right\} \le 1 - \nu_{{t_{k} }}^{3} \le 1 - \mathop {\min }\limits_{{t_{k} }} \left\{ {\nu_{{t_{k} }}^{3} } \right\} \hfill \\ \Rightarrow \mathop \prod \limits_{k = 1}^{p} \left( {1 - \mathop {\max }\limits_{{t_{k} }} \left\{ {\nu_{{t_{k} }}^{3} } \right\}} \right)^{{\epsilon_{{t_{k} }} }} \le \mathop \prod \limits_{k = 1}^{p} \left( {1 - \nu_{{t_{k} }}^{3} } \right)^{{\epsilon_{{t_{k} }} }} \le \mathop \prod \limits_{k = 1}^{p} \left( {1 - \mathop {\min }\limits_{{t_{k} }} \left\{ {\nu_{{t_{k} }}^{3} } \right\}} \right)^{{\epsilon_{{t_{k} }} }} \hfill \\ \Rightarrow \left( {1 - \mathop {\max }\limits_{{t_{k} }} \left\{ {\nu_{{t_{k} }}^{3} } \right\}} \right)^{{\mathop \sum \limits_{k = 1}^{p} \epsilon_{{t_{k} }} }} \le \mathop \prod \limits_{k = 1}^{p} \left( {1 - \nu_{{t_{k} }}^{3} } \right)^{{\epsilon_{{t_{k} }} }} \le \left( {1 - \mathop {\min }\limits_{{t_{k} }} \left\{ {\nu_{{t_{k} }}^{3} } \right\}} \right)^{{\mathop \sum \limits_{k = 1}^{p} \epsilon_{{t_{k} }} }} \hfill \\ \Rightarrow \left( {1 - \mathop {\max }\limits_{{t_{k} }} \left\{ {\nu_{{t_{k} }}^{3} } \right\}} \right) \le \mathop \prod \limits_{k = 1}^{p} \left( {1 - \nu_{{t_{k} }}^{3} } \right)^{{\epsilon_{{t_{k} }} }} \le \left( {1 - \mathop {\min }\limits_{{t_{k} }} \left\{ {\nu_{{t_{k} }}^{3} } \right\}} \right) \hfill \\ \Rightarrow \mathop {\min }\limits_{{t_{k} }} \left\{ {\nu_{{t_{k} }}^{3} } \right\} \le 1 - \mathop \prod \limits_{k = 1}^{p} \left( {1 - \nu_{{t_{k} }}^{3} } \right)^{{\epsilon_{{t_{k} }} }} \le \mathop {\max }\limits_{{t_{k} }} \left\{ {\nu_{{t_{k} }}^{3} } \right\} \hfill \\ \Rightarrow \sqrt[3]{{\mathop {\min }\limits_{{t_{k} }} \left\{ {\nu_{{t_{k} }}^{3} } \right\} }} \le \sqrt[3]{{1 - \mathop \prod \limits_{k = 1}^{p} \left( {1 - \nu_{{t_{k} }}^{3} } \right)^{{\epsilon_{{t_{k} }} }} }} \le \sqrt[3]{{\mathop {\max }\limits_{{t_{k} }} \left\{ {\nu_{{t_{k} }}^{3} } \right\}}} \hfill \\ \Rightarrow \mathop {\min }\limits_{{t_{k} }} \left\{ {\nu_{{t_{k} }} } \right\} \le \nu_{t} \le \mathop {\max }\limits_{{t_{k} }} \left\{ {\nu_{{t_{k} }} } \right\} \hfill \\ \end{gathered}$$

Hence by comparing relation 5 and 6, we obtain that

$${{F}_{t}}^{-}\le FFDyWG\left({F}_{{t}_{1}}, {F}_{{t}_{2}},..., {F}_{{t}_{p}}\right)\le {{F}_{t}}^{+}$$

#### Theorem 8

(Monotonicity) Consider two collections $p$ numbers of FFNs, denoted as ${F}_{{t}_{k}}=\left({\mu }_{{t}_{k}}, {\upnu }_{{{\text{t}}}_{{\text{k}}}}\right)$ and ${G}_{{t}_{k}}=\left({\eta }_{{t}_{k}}, {\xi }_{{t}_{k}}\right)$, with $k$ ranging from $1$ to $p$. Let ${\epsilon }_{t}={[{\epsilon }_{{t}_{1}},{\epsilon }_{{t}_{2}},...,{\epsilon }_{{t}_{p}}] }^{T}$ be the weight vector corresponding to ${t}_{k}$, where $k=\mathrm{1,2}, \dots , p$, such that ${\epsilon }_{{t}_{k}}\in \left[\mathrm{0,1}\right]$ and ${\sum }_{k=1}^{p}{\epsilon }_{{t}_{k}}=1$. If for each ${t}_{k}$: ${\mu }_{{t}_{k}}\le {\eta }_{{t}_{k}}$ and ${\nu }_{{t}_{k}}\ge {\xi }_{{t}_{k}}$, then:

$$FFDyWG\left({F}_{{t}_{1}}, {F}_{{t}_{2}},..., {F}_{{t}_{p}}\right)\le FFDyWG\left({G}_{{t}_{1}}, {G}_{{t}_{2}}, ... , {G}_{{t}_{p}}\right).$$

#### Proof

The proof of this theorem is omitted because it is identical to that of Theorem 4.

## Application of proposed FF dynamic weighted aggregation operators in MADM problem

In this section, we introduce a novel approach to address MADM problems using FF information through the application of FF Dynamic weighted aggregation operators.

- Let us consider a discrete set of alternatives denoted as $\left\{{\Xi }_{1}, {\Xi }_{2}, ... ,{\Xi }_{m}\right\}$.
- Consider a set of attributes $\left\{{\varnothing }_{1}, {\varnothing }_{2}, ... ,{\varnothing }_{n}\right\}$, each associated with a weight vector $\omega ={\left[{\omega }_{1}, {\omega }_{2},...,{\omega }_{n}\right]}^{T}$, where ${\omega }_{j}\ge 0$ for $j = 1, 2, ..., n$, and ${\sum }_{j=1}^{n}{\omega }_{j}=1$.
- We further include the concept of different time periods ${t}_{k}$, where $k=\mathrm{1,2}, \dots , p$. Each time period is characterized by a weight vector ${\epsilon }_{t}={\left[{\epsilon }_{{t}_{1}}, {\epsilon }_{{t}_{2}} ... {\epsilon }_{{t}_{p}}\right]}^{T}$, with ${\epsilon }_{{t}_{k}}\in \left[\mathrm{0,1}\right]$ and ${\sum }_{k=1}^{p}{\epsilon }_{{t}_{k}}=1$.
- Let ${R}_{{t}_{k}}=[{{o}_{ij\left({t}_{k}\right)}]}_{m \times n}={\left({\mu }_{ij\left({t}_{k}\right)}, {\nu }_{ij\left({t}_{k}\right)}\right)}_{m \times n}$ represent the FF decision matrices for time period ${t}_{k}$, where ${\mu }_{ij\left({t}_{k}\right)}$ indicates the degree to which alternative ${\Xi }_{i}$ satisfies attribute ${\varnothing }_{j}$ during time periods ${t}_{k}$, and ${\nu }_{ij\left({t}_{k}\right)}$ signifies the degree to which alternative ${\Xi }_{i}$ fails to satisfy attribute ${\varnothing }_{j}$ at time periods ${t}_{k}$. These values are compliant with the constraints: ${\mu }_{ij\left({t}_{k}\right)}\in \left[\mathrm{0,1}\right]$, ${\nu }_{ij\left({t}_{k}\right)}\in \left[\mathrm{0,1}\right]$ and ${{(\mu }_{ij\left({t}_{k}\right)})}^{3}+{{(\nu }_{ij\left({t}_{k}\right)})}^{3}\le 1$.

Now, utilizing the previously presented decision information, we developed an efficient algorithm to decide on and rank the most preferable alternative(s) within the context of MADM.

### Procedure for FFDyWA

*Step 1* Utilize the FFDyWA operator:

$${o}_{ij\left({t}_{k}\right)}=\left({\mu }_{ij\left({t}_{k}\right)}, {\nu }_{ij\left({t}_{k}\right)}\right)=FFDyWA\left({o}_{ij\left({t}_{1}\right)}, {o}_{ij\left({t}_{2}\right)},..., {o}_{ij\left({t}_{p}\right)}\right)$$

This implies that

$${o}_{ij\left({t}_{k}\right)}=\left(\sqrt[3]{1-{\prod }_{k=1}^{p}{\left(1-{\mu }_{ij({t}_{k})}^{3}\right)}^{{\epsilon }_{{t}_{k}}}} , {\prod }_{k=1}^{p}{\nu }_{ij\left({t}_{k}\right)}^{{\epsilon }_{{t}_{k}}}\right)$$

where, $i=\mathrm{1,2},... ,m$ and , $j=\mathrm{1,2},... ,n$. This process aggregates all the FF decision matrices into a collective FF decision matrix as follows ${R}_{t}=[{{o}_{ij}]}_{m \times n}= {\left({\mu }_{ij}, {\nu }_{ij}\right)}_{m \times n}$.

*Step 2.* Apply the FFWA operator as follows:

$${o}_{i}=\left({\mu }_{i},{\nu }_{i}\right)=FFWA\left({o}_{i1}, {o}_{i2}, ... , {o}_{in}\right)=\left( \sqrt[3]{1-{\prod }_{j=1}^{n}{\left(1-{\mu }_{ij}^{3}\right)}^{{\omega }_{j}}} , {\prod }_{j=1}^{n}{\nu }_{ij}^{{\omega }_{j}}\right).$$

This step yields the overall values ${o}_{i}=\left({\mu }_{i},{\nu }_{i}\right)$ of the alternatives ${\Xi }_{i}$, where $i=\mathrm{1,2},... , m$.

*Step 3.* Compute the scores values ${o}_{i}$ corresponding to each alternatives ${\Xi }_{i}$ by using Definition 4.

*Step 4.* Arrange all the alternatives ${\Xi }_{i}$, for $i=\mathrm{1,2},... , m$, and identify the optimal choice(s) using $g({o}_{i})$.

**Figure 2:**
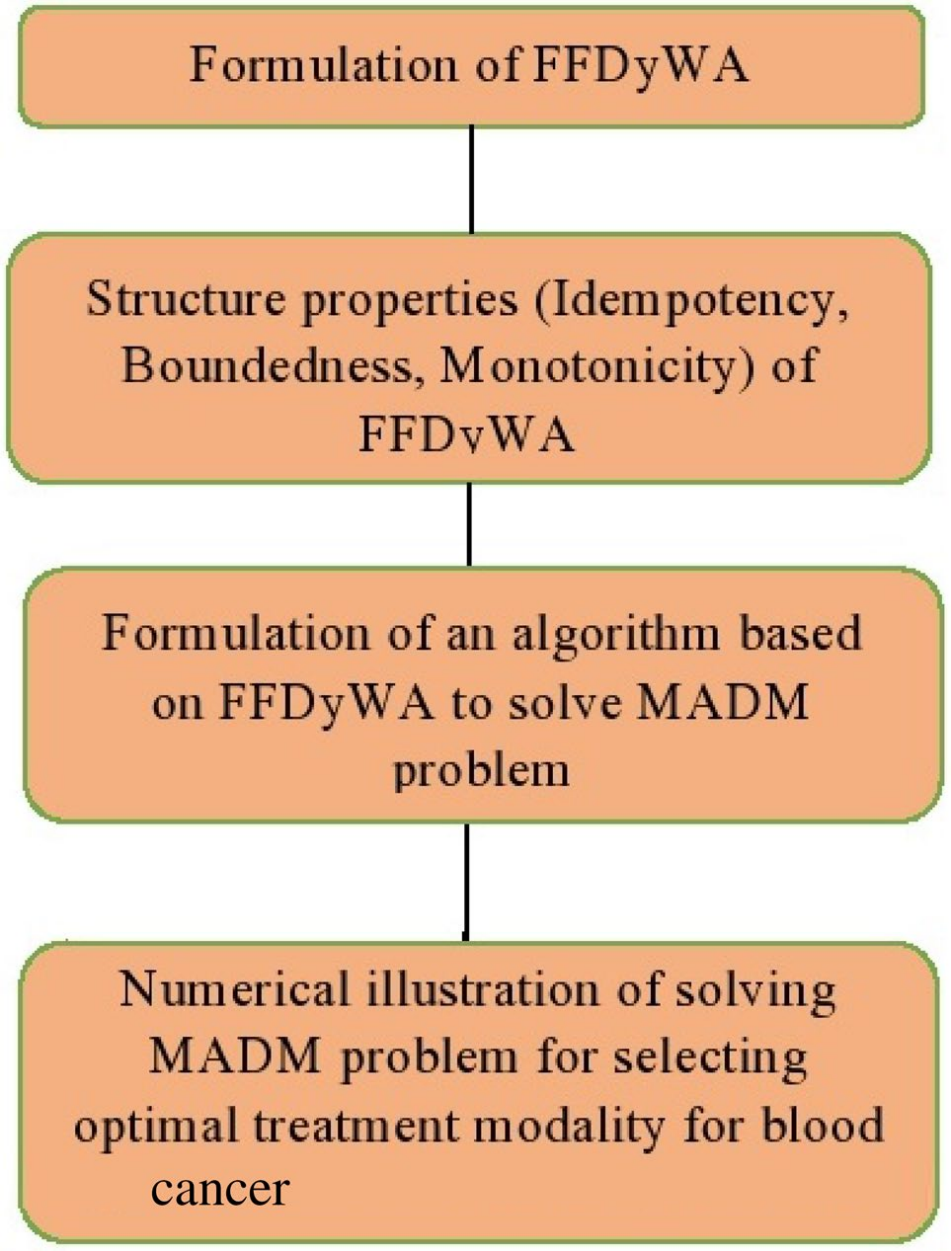
Flowchart of the proposed algorithm under FFDyWA.

The graphical representation of the aforementioned steps of the proposed algorithm utilizing FFDyWA is shown in Fig. 2.

### Procedure for FFDyWG

*Step 1.* Utilize the FFDyWG operator, represented as follows:

$${o}_{ij\left({t}_{k}\right)}=\left({\mu }_{ij\left({t}_{k}\right)},{\nu }_{ij\left({t}_{k}\right)}\right)= FFDyWG\left({o}_{ij\left({t}_{1}\right)}, {o}_{ij\left({t}_{2}\right)},..., {o}_{ij\left({t}_{p}\right)}\right)$$

This implies that

$${o}_{ij\left({t}_{k}\right)}=\left({\prod }_{k=1}^{p}{\mu }_{ij\left({t}_{k}\right)}^{{\epsilon }_{{t}_{k}}} , \sqrt[3]{1-{\prod }_{k=1}^{p}{\left(1-{\nu }_{ij({t}_{k})}^{3}\right)}^{{\epsilon }_{{t}_{k}}}}\right)$$

where, $i=\mathrm{1,2},... ,m$ and , $j=\mathrm{1,2},... ,n$. This operator is employed to aggregates all the FF decision matrices into a collective FF decision matrix as follows ${R}_{t}=[{{o}_{ij}]}_{m \times n}= {\left({\mu }_{ij}, {\nu }_{ij}\right)}_{m \times n}$.

*Step 2.* Apply the FFWG operator, defined as:

$${o}_{i}=\left({\mu }_{i},{\nu }_{i}\right)=FFWG\left({o}_{i1}, {o}_{i2}, ... , {o}_{in}\right)=\left({\prod }_{j=1}^{n}{\mu }_{ij}^{{\omega }_{j}} , \sqrt[3]{1-{\prod }_{j=1}^{n}{\left(1-{\nu }_{ij}^{3}\right)}^{{\omega }_{j}}}\right).$$

This step yields the overall values ${o}_{i}=\left({\mu }_{i},{\nu }_{i}\right)$ for the alternatives ${\Xi }_{i}$, where $i=\mathrm{1,2},... , m$.

*Step 3.* Compute the scores values ${o}_{i}$ corresponding to each alternatives ${\Xi }_{i}$ by using Definition 4.

*Step 4.* Rank all the alternatives ${\Xi }_{i}$ (where $i=\mathrm{1,2},... , m$) and select the best one(s) in accordance with the criteria of $g({o}_{i})$.

**Figure 3:**
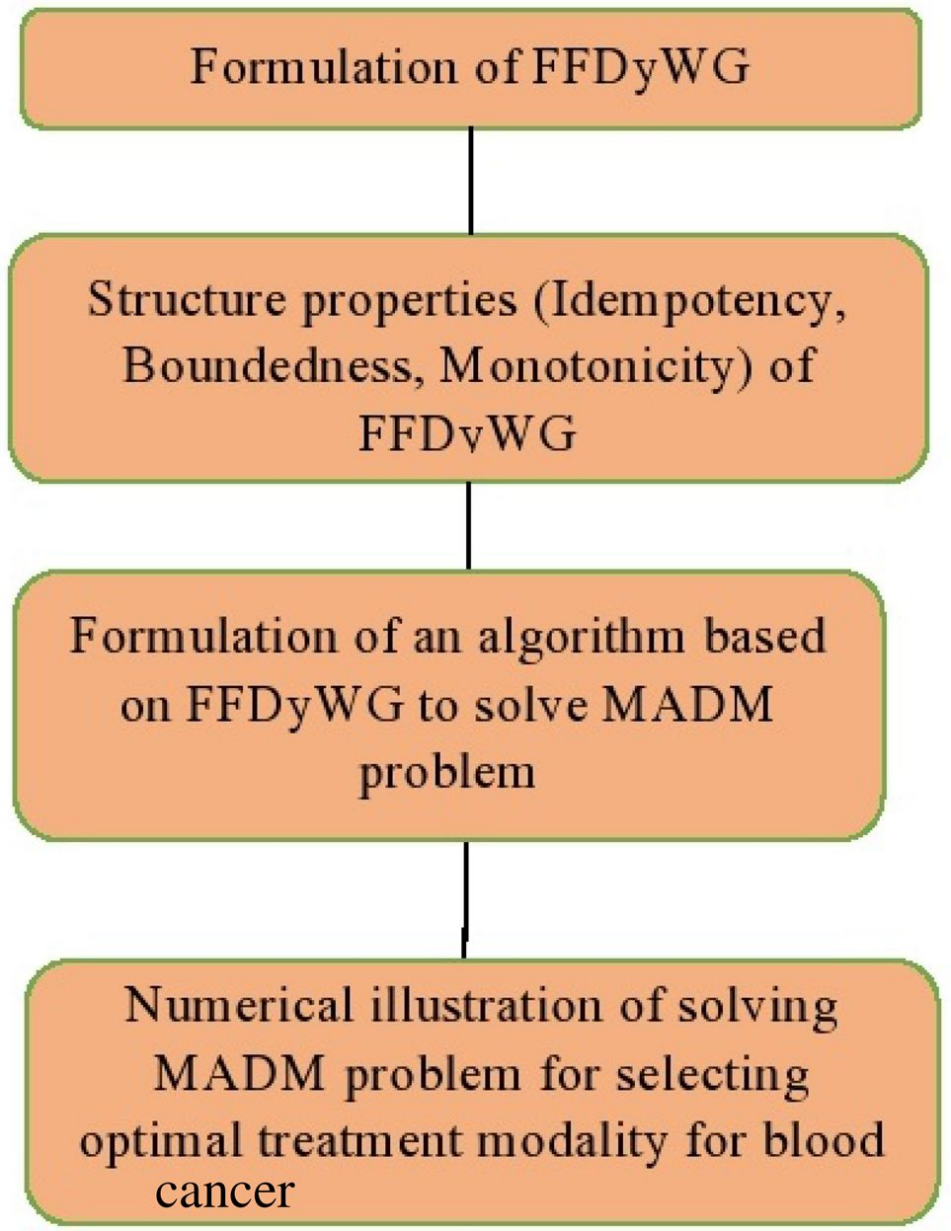
Flowchart of the proposed algorithm under FFDyWG.

The graphical representation of the aforementioned steps of the proposed algorithm utilizing FFDyWA is shown in Fig. 3.

Both proposed schemes are very handy in solving MADM problems in the context of road safety measures, dynamic financial strategies, medicines, education, big data analytics, energy resources, and many more. In this article, we consider the following decision-making problem of treatment methods to cure blood cancer as an illustrated example to showcase the practicability of the proposed strategies.

## An optimal treatment modality to cure blood cancer under FF dynamic environment

In this section, we effectively apply the proposed strategies of this article to obtain an optimal treatment modality to cure blood cancer under FF Dynamic knowledge.

Cancer is a major cause of death, causing the uncontrolled proliferation of abnormal cells in the body, leading to cellular dysfunction or death. Blood cancer, a form of neoplastic disorders, affects blood cells and is primarily caused by genetic mutations. Leukemia, a hematologic malignancy, is distinguished by the unrestrained proliferation of malignant leukocytes within the bone marrow. Clinical manifestations include hemorrhagic tendencies, musculoskeletal discomfort, asthenia, pyrexia, and increased susceptibility to infectious pathogens. The cause is unknown, but it is thought to be a combination of genetic and non-inherited environmental elements. Risk factors for developing certain conditions include smoking, exposure to ionizing radiation, petrochemicals, previous chemotherapy treatment, and Down syndrome. There are four major categories of leukemia: acute lymphoblastic leukemia (ALL), chronic lymphocytic leukemia (CLL), chronic myeloid leukemia (CML), acute myeloid leukemia (AML), and several rarer variants. Hematopoietic stem cell transplantation (HSCT) is a unique procedure but may provide complications. In the consolidation phase, targeted drugs like Imatinib mesylate and standard chemotherapy have shown to improve survival rates.

Following are the treatment approaches for ALL disease.

### Chimeric antigen receptor (CAR) T-cells therapy

Advancements in the employment of CAR T-cell therapy have significantly evolved, emerging as an increasingly pivotal modality for addressing hematological malignancies^53^. Kymriah and Yescarta, the inaugural class of CAR T-cell therapeutics, obtained approval from the Food and Drug Administration in the United States for the treatment of leukemia and lymphoma, in August and October of 2017, respectively^54^. CAR T-cells denote T or natural killer cells that have been subjected to genetic manipulation, resulting in the expression of hybrid proteins. These amalgamated proteins function as navigational cues, steering the engineered cells towards precise molecular targets located on the surface of neoplastic cells. CAR T-cell constructs comprise an extracellular antigen recognition moiety, namely the single-chain variable fragment (scFv) derived from an antibody, an integral transmembrane domain, and an intracellular signaling domain. The targeting moiety is inherently supplied in its native form within the groove of major histocompatibility complex (MHC) molecules, without requiring any supplementary processing. CAR T-cells have the ability to recognize and target tumor cells without being influenced by the MHC haplotype of the patient.

The utilization of ligands or peptides for the purpose of targeting CAR T-cell therapy is a field of active advancement and investigation. This paper discusses the various functions and applications of monoclonal antibodies, specifically focusing on chimeric antibodies. CAR-T-cell and other targeted therapies for adult ALL are still being studied. These treatments in combination or sequentially may improve adult ALL cure rates to those of juvenile ALL. This method may also reduce the need for intensive and maintenance chemotherapy.

### Chemotherapy

Paul Ehrlich^55,56^, a German chemist with a focus on alkylating compounds, coined the term "chemotherapy" to denote the chemical intervention in the management of diseases. Chemotherapy pertains to the administration of pharmacological agents for the explicit purpose of treating cancer. These chemotherapeutic agents are delivered systemically through the circulatory system to effectively target neoplastic cells distributed throughout the entire organism. Chemotherapy exhibits notable efficacy in the management of malignancies, particularly leukemia, which has disseminated extensively within the organism. Certain advanced forms of malignancies, such as acute lymphoblastic and acute myelogenous leukemia, are amenable to curative treatment through chemotherapy. While curative outcomes are not uniformly achieved in the treatment of these malignancies, significant progress has been made in improving progression-free survival and overall survival rates. Chemotherapy is commonly linked to off-target effects, resulting in a detrimental impact on neighboring healthy cells. These effects can manifest in various forms, including but not limited to hair loss, nausea, vomiting, fatigue, mouth sores, hand or foot rashes, diarrhea, and impaired liver or kidney function. The observed negative effects can be attributed to excessive medication administration^57,58^.

### Radiation therapy

Radiation therapy constitutes an essential component of cancer treatment. At some stage, a majority of cancer patients, over 50%, require radiation therapy. The number of individuals who have survived cancer in the long term has experienced a substantial increase due to advancements in treatment approaches. Individuals who have survived cancer for an extended period of time have a heightened susceptibility to the development of subsequent cancers. Radiation therapy has been utilized for the purpose of CNS prophylaxis and for the management of certain instances of extramedullary recurrence. Cranial and/or craniospinal irradiation have been recognized as the most longstanding CNS prophylactic treatments for individuals diagnosed with ALL, including both children and adult populations^59^. In the treatment of ALL, radiation therapy is occasionally employed in conjunction with chemotherapy. In cases where chemotherapy proves ineffective, radiation treatment can serve as a viable intervention to impede the proliferation of leukemia cells inside the bone marrow. The eradication of cancer cells has the potential to alleviate bone discomfort resulting from the accumulation of cells associated with ALL. Radiation treatment has demonstrated efficacy in treating CNS in ALL; nonetheless, it is frequently accompanied by delayed side effects, including secondary neoplasms, endocrinopathy, neurocognitive impairment, and neurotoxicity^60^. From a clinical and, to a certain extent, biological perspective, it is imperative to establish a clear differentiation between early and late adverse effects. The initial effects become apparent within a short period of time following the conclusion of a fractionated radiation treatment regimen. The aforementioned consequences encompass cutaneous erythema, both dry and wet desquamation of the skin, mucositis, as well as symptoms of nausea and diarrhea. The manifestation of late effects is commonly observed following extended durations of latency, spanning from several months to several years. These effects encompass radiation-induced fibrosis, atrophy, vascular impairment, and brain impairment, as well as a variety of endocrine and growth-related consequences^61^.

### Hematopoietic stem-cell transplantation

Hematopoietic stem cell transplantation (HSCT) functions as a therapeutic approach aimed at consolidating the treatment of acute leukemia, resulting in the potential for cure in a significant number of patients with relapsed or very aggressive disease. The therapeutic efficacy of HSCT is a result of the combined action of direct cytotoxicity induced by the chemo-radiotherapy provided during the conditioning regimen, as well as an immune-mediated impact known as graft-versus-leukemia (GVL). Patients who undergo transplantation with a lower illness burden tend to experience more favorable outcomes^62^. Furthermore, it has been shown that individuals suffering from mild to severe GVHD have elevated rates of sustained remission over an extended period. This phenomenon is believed to be a result of an immunological reaction directed towards the host's own tissues^63,64^. The primary mediators of GVL response in ALL are T cells; however, the specific antigens recognized by these allogeneic T cells are mostly unidentified. The loss of antigen presentation mechanisms has been identified as a well-documented factor contributing to leukemic resistance against the GVL impact^65^.

In the forthcoming analysis, we undertake a comparative assessment of CAR-T cell therapy vis-à-vis established conventional modalities for the treatment of ALL by employing an analytical MADM technique, specifically under Fermatean fuzzy decision-making. Our approach involves the incorporation of CAR-T cell therapy into the comparative framework alongside the adaptation of evaluation criteria, comparative methodologies, and ranking procedures.

### Illustration

Here, we delineate a systematic procedure for the evaluation and selection of optimal treatment alternatives for ALL within the framework of FF Dynamic aggregation operators.

Let us denote the set of treatment alternatives as $\left\{{\Xi }_{1},{\Xi }_{2},{\Xi }_{3},{\Xi }_{4}\right\}$, where

i. $${\Xi }_{1}:$$ Signifies Chemotherapy
ii. ${\Xi }_{2}:$ Represents Hematopoietic Stem-Cell Transplantation (HSCT)
iii. ${\Xi }_{3}:$ Denotes Radiation therapy
iv. ${\Xi }_{4}$: Corresponds to Chimeric Antigen Receptor (CAR) T-cell Therapy

Furthermore, we identify a set of pertinent attributes as $\left\{{\varnothing }_{1},{\varnothing }_{2},{\varnothing }_{3},{\varnothing }_{4},{\varnothing }_{5}\right\}$ each contributing to the assessment of treatment options:

i. $${\varnothing }_{1}:$$ Survival Rate
ii. ${\varnothing }_{2}:$ Achieving Remission (The primary goal of ALL treatment is to induce remission, which means that leukemia cells are no longer detectable in the blood or bone marrow. Achieving remission is a critical milestone in the treatment of ALL).
iii. ${\varnothing }_{3}:$ Side Effects
iv. $${\varnothing }_{4}:$$ Efficiency
v. $${\varnothing }_{5}:$$ Reliability

The four possible alternatives ${\Xi }_{i}$, will be evaluated using the FF information by the decision-maker under the attributes ${\varnothing }_{j}$ at three different periods ${t}_{k}$, where ${t}_{1},{t}_{2},$ and ${t}_{3}$ represent periods of $2017-2019$, $2019-2021$ and $2021-2023$, respectively. The weight vectors for the periods and attributes are denoted as ${\epsilon }_{t}={[0.2, \mathrm{0.3,0.5}]}^{T}$ and $\omega {=\left[0.3, 0.25, 0.1, \mathrm{0.2,0.15}\right] }^{T}$, respectively. The decision matrices ${R}_{{t}_{1}},{R}_{{t}_{2}}$ and ${R}_{{t}_{3}}$ are presented in Tables 1, 2 and 3 respectively.

**Table 1:** Decision Matrix ${R}_{{t}_{1}}$.

	${\varnothing }_{1}$	${\varnothing }_{2}$	${\varnothing }_{3}$	${\varnothing }_{4}$	${\varnothing }_{5}$
${\Xi }_{1}$	$\left(\mathrm{0.7,0.4}\right)$	$\left(\mathrm{0.6,0.5}\right)$	$\left(\mathrm{0.9,0.6}\right)$	$\left(\mathrm{0.8,0.4}\right)$	$\left(\mathrm{0.7,0.3}\right)$
${\Xi }_{2}$	$\left(\mathrm{0.8,0.6}\right)$	$\left(\mathrm{0.7,0.8}\right)$	$\left(\mathrm{0.7,0.4}\right)$	$\left(\mathrm{0.7,0.6}\right)$	$\left(\mathrm{0.6,0.6}\right)$
${\Xi }_{3}$	$\left(\mathrm{0.6,0.8}\right)$	$\left(\mathrm{0.5,0.7}\right)$	$\left(\mathrm{0.8,0.5}\right)$	$\left(\mathrm{0.7,0.5}\right)$	$\left(\mathrm{0.5,0.4}\right)$
${\Xi }_{4}$	$\left(\mathrm{0.9,0.6}\right)$	$\left(\mathrm{0.8,0.4}\right)$	$\left(\mathrm{0.5,0.8}\right)$	$\left(\mathrm{0.6,0.4}\right)$	$\left(\mathrm{0.7,0.5}\right)$

**Table 2:** Decision matrix ${R}_{{t}_{2}}$.

	${\varnothing }_{1}$	${\varnothing }_{2}$	${\varnothing }_{3}$	${\varnothing }_{4}$	${\varnothing }_{5}$
${\Xi }_{1}$	$\left(\mathrm{0.6,0.7}\right)$	$\left(\mathrm{0.7,0.6}\right)$	$\left(\mathrm{0.8,0.6}\right)$	$\left(\mathrm{0.8,0.7}\right)$	$\left(\mathrm{0.7,0.5}\right)$
${\Xi }_{2}$	$\left(\mathrm{0.7,0.6}\right)$	$\left(\mathrm{0.8,0.7}\right)$	$\left(\mathrm{0.6,0.5}\right)$	$\left(\mathrm{0.6,0.9}\right)$	$\left(\mathrm{0.6,0.5}\right)$
${\Xi }_{3}$	$\left(\mathrm{0.5,0.7}\right)$	$\left(\mathrm{0.6,0.6}\right)$	$\left(\mathrm{0.7,0.5}\right)$	$\left(\mathrm{0.7,0.6}\right)$	$\left(\mathrm{0.5,0.6}\right)$
${\Xi }_{4}$	$\left(\mathrm{0.7,0.5}\right)$	$\left(\mathrm{0.7,0.5}\right)$	$\left(\mathrm{0.4,0.7}\right)$	$\left(\mathrm{0.7,0.4}\right)$	$\left(\mathrm{0.8,0.6}\right)$

**Table 3:** Decision Matrix ${R}_{{t}_{3}}$.

	${\varnothing }_{1}$	${\varnothing }_{2}$	${\varnothing }_{3}$	${\varnothing }_{4}$	${\varnothing }_{5}$
${\Xi }_{1}$	$\left(\mathrm{0.7,0.5}\right)$	$\left(\mathrm{0.7,0.5}\right)$	$\left(\mathrm{0.9,0.4}\right)$	$\left(\mathrm{0.8,0.5}\right)$	$\left(\mathrm{0.8,0.4}\right)$
${\Xi }_{2}$	$\left(\mathrm{0.8,0.7}\right)$	$\left(\mathrm{0.8,0.5}\right)$	$\left(\mathrm{0.7,0.5}\right)$	$\left(\mathrm{0.7,0.6}\right)$	$\left(\mathrm{0.7,0.8}\right)$
${\Xi }_{3}$	$\left(\mathrm{0.6,0.5}\right)$	$\left(\mathrm{0.6,0.4}\right)$	$\left(\mathrm{0.8,0.6}\right)$	$\left(\mathrm{0.6,0.4}\right)$	$\left(\mathrm{0.6,0.5}\right)$
${\Xi }_{4}$	$\left(\mathrm{0.9,0.5}\right)$	$\left(\mathrm{0.8,0.5}\right)$	$\left(\mathrm{0.3,0.4}\right)$	$\left(\mathrm{0.9,0.5}\right)$	$\left(\mathrm{0.8,0.5}\right)$

The presented MADM problem is addressed within the framework of the FFDyWA and FFDyWG operators. We outline the methodology and results of two distinct approaches, Method I and Method II, for resolving this complex decision problem.

#### Method I (FFDyWA operator)

*Step 1.* The FFDyWA operator is applied to aggregate the FF decision matrices ${R}_{{t}_{1}},{R}_{{t}_{2}}$ and ${R}_{{t}_{3}}$, yielding a collective FF decision matrix ${R}_{t}$. The calculated values are displayed in Table 4.

**Table 4:** Collective matrix ${R}_{t}$ using FFDyWA operator.

	${\varnothing }_{1}$	${\varnothing }_{2}$	${\varnothing }_{3}$	${\varnothing }_{4}$	${\varnothing }_{5}$
${\Xi }_{1}$	$\left(\mathrm{0.674,0.528}\right)$	$\left(\mathrm{0.683,0.528}\right)$	$\left(\mathrm{0.877,0.489}\right)$	$\left(\mathrm{0.8,0.528}\right)$	$\left(\mathrm{0.756,0.403}\right)$
${\Xi }_{2}$	$\left(\mathrm{0.775,0.648}\right)$	$\left(\mathrm{0.784,0.607}\right)$	$\left(\mathrm{0.674,0.478}\right)$	$\left(\mathrm{0.647,0.677}\right)$	$\left(\mathrm{0.656,0.655}\right)$
${\Xi }_{3}$	$\left(\mathrm{0.574,0.607}\right)$	$\left(\mathrm{0.583,0.505}\right)$	$\left(\mathrm{0.775,0.547}\right)$	$\left(\mathrm{0.656,0.472}\right)$	$\left(\mathrm{0.555,0.505}\right)$
${\Xi }_{4}$	$\left(\mathrm{0.864,0.518}\right)$	$\left(\mathrm{0.775,0.478}\right)$	$\left(\mathrm{0.388,0.543}\right)$	$\left(\mathrm{0.825,0.447}\right)$	$\left(\mathrm{0.784,0.528}\right)$

*Step 2.* Subsequently, the FFWA operator is employed to derive the comprehensive values ${o}_{i}$ of ${\Xi }_{i}$, where $i=\mathrm{1,2},\mathrm{3,4}$.

$${o}_{1}=\left(0.749, 0.503\right)$$

$${o}_{2}=\left(0.725, 0.624\right)$$

$${o}_{3}= \left(\mathrm{0.621,0.530}\right)$$

$${o}_{4}= \left(\mathrm{0.804,0.496}\right)$$

*Step 3.* Calculate the scores $g{(o}_{i})$ where $i=\mathrm{1,2},\mathrm{3,4}$ of the overall FF preferences values ${o}_{i}$ to rank all the alternative ${\Xi }_{i}$:

$$g{(o}_{1})=0.292 \, g{(o}_{2})=0.138$$

$$g{(o}_{3})=0.090 \, g{(o}_{4})=0.397$$

*Step 4.* The ranking order of the alternatives is established, revealing that ${\Xi }_{4}\succ {\Xi }_{1}\succ {\Xi }_{2}\succ {\Xi }_{3}$. Consequently, CAR T-cell therapy is identified as the optimal choice.

**Figure 4:**
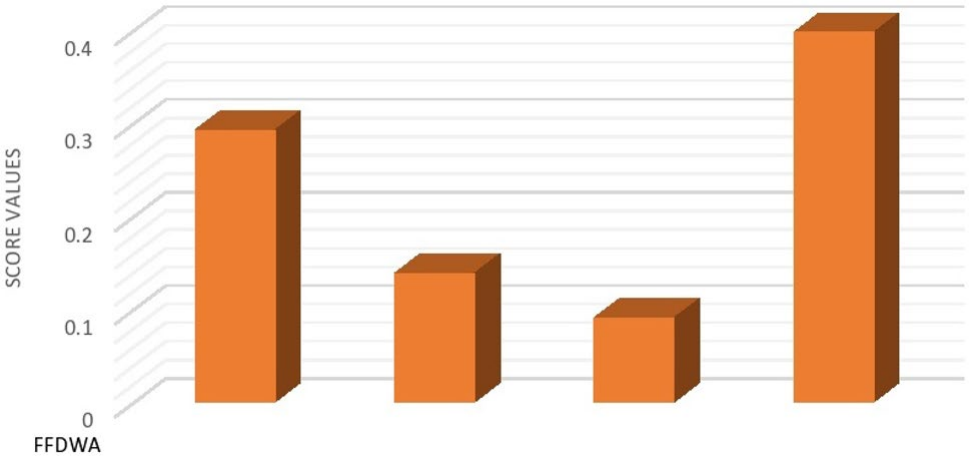
Ranking of alternatives using FFDyWA.

The above procedure is graphically depicted in Fig. 4, which represents the score values of the alternatives obtained from FFDyWA operator.

Similarly, in accordance with the FFDyWG operator framework, the resolution of the aforementioned MADM problem is executed as follows:

#### Method II (FFDyWG operator)

*Step 1.* Employ the FFDyWG operator to amalgamate all FF decision matrices, denoted $({t}_{k})$ into a unified FF decision matrix ${R}_{t}$. These details are summarized as FFNs in Table 5 as follows:

**Table 5:** Collective matrix ${R}_{t}$ under FFDyWG operator.

	${\varnothing }_{1}$	${\varnothing }_{2}$	${\varnothing }_{3}$	${\varnothing }_{4}$	${\varnothing }_{5}$
${\Xi }_{1}$	$\left(\mathrm{0.668,0.571}\right)$	$\left(\mathrm{0.678,0.535}\right)$	$\left(\mathrm{0.868,0.523}\right)$	$\left(\mathrm{0.8,0.571}\right)$	$\left(\mathrm{0.748,0.411}\right)$
${\Xi }_{2}$	$\left(\mathrm{0.768,0.656}\right)$	$\left(\mathrm{0.778,0.658}\right)$	$\left(\mathrm{0.668,0.483}\right)$	$\left(\mathrm{0.668,0.754}\right)$	$\left(\mathrm{0.648,0.711}\right)$
${\Xi }_{3}$	$\left(\mathrm{0.568,0.658}\right)$	$\left(\mathrm{0.578,0.557}\right)$	$\left(\mathrm{0.768,0.555}\right)$	$\left(\mathrm{0.648,0.499}\right)$	$\left(\mathrm{0.547,0.521}\right)$
${\Xi }_{4}$	$\left(\mathrm{0.834,0.524}\right)$	$\left(\mathrm{0.768,0.483}\right)$	$\left(\mathrm{0.362,0.639}\right)$	$\left(\mathrm{0.769,0.456}\right)$	$\left(\mathrm{0.778,0.535}\right)$

*Step 2.* Apply the FFWG operator to derive the comprehensive values ${o}_{i}$ of ${\Xi }_{i}$, wherer $i=\mathrm{1,2},\mathrm{3,4}$.

$${o}_{1}=\left(0.725, 0.539\right)$$

$${o}_{2}=\left(0.720, 0.678\right)$$

$${o}_{3}= \left(\mathrm{0.600,0.579}\right)$$

$${o}_{4}= \left(\mathrm{0.731,0.520}\right)$$

*Step 3.* Calculate the scores $g{(o}_{i})$ wherer $i=\mathrm{1,2},\mathrm{3,4}$ of the overall FF preferences values ${o}_{i}$ to rank all the alternative ${\Xi }_{i}$:

$$g{(o}_{1})=0.224 \, g{(o}_{2})=0.061$$

$$g{(o}_{3})=0.020 \, g{(o}_{4})=0.250$$

*Step 4.* Consequently, the ranking order of the provided alternatives is established as follows: ${\Xi }_{4}\succ {\Xi }_{1}\succ {\Xi }_{2}\succ {\Xi }_{3}$. Therefore, CAR T-cell therapy emerges as the optimal alternative.

**Figure 5:**
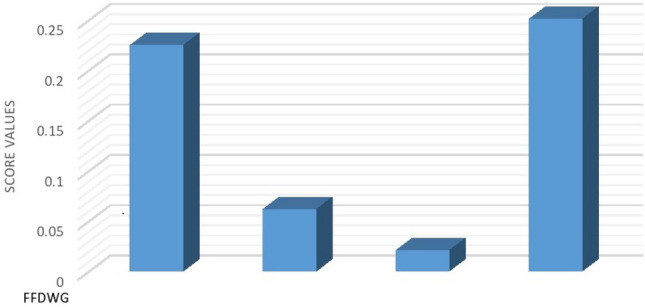
Ranking of alternatives using FFDyWG.

The above procedure is graphically depicted in Fig. 5, which indicate the score values of the alternatives obtain from FFDyWG operator.

The preceding explanation demonstrates that CAR T-cells therapies are the optimal treatment for ALL. The following are some advantages of CAR T-cell therapies: CAR T-cell therapies offer numerous benefits over conventional treatments. These can induce long-term, complete remissions in multiple-treatment-resistant cancer patients. Additionally, the treatment duration is shorter, allowing for a more efficient and streamlined therapeutic process. Moreover, CAR T-cell therapies facilitate a rapid recovery, enabling patients to regain their health and well-being more quickly than with conventional treatments. These also benefit from the utilization of viable cells, which possess the capacity to proliferate within the bodies of patients, thereby establishing a long-lasting immunological memory. Hence, the presence of persistent CAR T-cells enables the identification and eradication of cancerous cells during instances of disease recurrence^66^.

### Comparative analysis

During this discourse, we undertake a comparative examination in order to assess the reliability of the suggested methodologies in relation to the established approaches outlined in^48^. We utilize two approaches, namely the IFDWA and IFDWG operators, to accumulate and combine identical data. The results produced through the utilization of these operators are compiled in Table 6 and arranged in Table 7 according to their ranking.

**Table 6:** Aggregated values of alternative obtained from different existing operators.

	IFDWA^48^	IFDWG^48^
$g\left({\Xi }_{1}\right)$	$\left(\mathrm{0.744,0.503}\right)$	$\left(\mathrm{0.725,0.525}\right)$
$g\left({\Xi }_{2}\right)$	$\left(\mathrm{0.734,0.624}\right)$	$\left(\mathrm{0.720,0.662}\right)$
$g\left({\Xi }_{3}\right)$	$\left(\mathrm{0.614,0.530}\right)$	$\left(\mathrm{0.600,0.560}\right)$
$g\left({\Xi }_{4}\right)$	$\left(\mathrm{0.793,0.496}\right)$	$\left(\mathrm{0.731,0.509}\right)$

**Table 7:** Ranking and score values for alternatives according to proposed and existing methodologies.

Methods	$S\left({r}_{1}\right)$	$S\left({r}_{2}\right)$	$S\left({r}_{3}\right)$	$S\left({r}_{4}\right)$	Ranking order
IFDWA^48^	$0.241$	$0.110$	$0.084$	$0.297$	${\Xi }_{4}\succ {\Xi }_{1}\succ {\Xi }_{2}\succ {\Xi }_{3}$
IFDWG^48^	$0.200$	$0.058$	$0.040$	$0.222$	${\Xi }_{4}\succ {\Xi }_{1}\succ {\Xi }_{2}\succ {\Xi }_{3}$
FFDyWA	$0.292$	$0.138$	$0.090$	$0.397$	${\Xi }_{4}\succ {\Xi }_{1}\succ {\Xi }_{2}\succ {\Xi }_{3}$
FFDyWG	$0.224$	$0.061$	$0.020$	$0.250$	${\Xi }_{4}\succ {\Xi }_{1}\succ {\Xi }_{2}\succ {\Xi }_{3}$

From the above discussion, it is quite evident that the methodologies developed in IF dynamic environment^48^ have less scope than the techniques presented in this article within the context of FF knowledge because the set of Intuitionistic membership degrees is less effective than the set of Fermatean membership degrees. It is, therefore, quite evident that dynamic FFS has a greater number of exhaustive options for identifying and resolving uncertainties than dynamic IFS.

The approaches developed in^25–34^ are not applicable for evaluating the information present in Tables 1, 2 and 3 because these techniques lack the ability to counter time-dependent decision-making problems. The efficacy of the proposed strategies in this study is enhanced due to their development within the framework of a dynamic FF environment because they take into account several time periods and enable a more precise evaluation of the information under consideration.

The theories of aggregation operators developed in the framework of the IF environment^15^ and PF knowledge^4^ contained a lot of restrictions because these strategies ignored the time periods, and due to this reason, they lost a lot of information and hence were unable to evaluate the information available in Tables 1, 2 and 3. While compared to them, the recently proposed techniques are more beneficial because these methodologies have been designed within the framework of dynamic FF knowledge equipped with a time interval, which has very accurately evaluated the considered information.

As a result, the mathematical designs of the proposed operator based on FFSs with time periods are powerful and comprehensive. This demonstrates the superiority of the developed techniques over other existing methods.

## Conclusions

This paper aims to present novel strategies for tackling decision-making problems in dynamic FF environment. The existing body of research has introduced several operators that have been shown to be helpful. However, it is worth noting that none of these operators have explicitly taken into account the time period in the FF setting. Because of this, using a dynamic FF model is a better way to express data about problems that change over time since it can handle bi-dimensional data within a unified framework in a better way. Considering these factors, we present a novel set of operators, namely FFDyWA and FFDyWG. We also examine the various characteristics of these operators.

Furthermore, we have introduced an innovative approach to address dynamic FF MADM problems. By utilizing the FFDyWG and FFDyWA operators, this strategy manages decision-related data pertaining to attribute values known as FFNs. Significantly, our methodology considers data gathered at various time intervals during the decision-making process in this dynamic setting.

Additionally, we present a practical demonstration of the application of these newly developed approaches in the selection of an optimal treatment for the treatment of blood cancer. It has been demonstrated that the suggested techniques have high efficacy in rapidly killing cancer, as time plays a crucial role in cancer treatment. Finally, a comparative study is performed to emphasize the importance and reliability of these innovative procedures in comparison to currently available techniques.

The proposed FF environment is superior to IFS and PFS as it encompasses the space of both IFS and PFS. The cubic sum of membership and non-membership degrees is bounded by 1 in the FF framework. Moreover, the proposed strategies are presented in dynamic FF framework which allows for data collection at various time intervals and therefore enables to handle uncertainties in more precise manner.

Although the proposed strategies enhance reliability with a better performance compared to the other existing techniques, it has some limitations, which include the following: (1) FFSs fails to handle the situation when cubic sum of membership and non-membership degrees exceed 1, and (2) it cannot be utilized to model cases involving spherical fuzzy information and picture fuzzy information because it only admits two parameters. These limitations will be addressed in our future work by applying the proposed strategies in the framework of q-rung fuzzy, picture and spherical fuzzy environment.

Future endeavors will primarily focus on developing a robust decision analysis tool, founded on dynamic operators, to enhance its practical utility and relevance. These approaches will be instrumental in shaping dynamic financial strategies, real-time social media monitoring, dynamic military management assessments, confidential short-listing in dynamic contexts, medical resource allocation for COVID-19, and intricate fuzzy dynamic decision-making paradigms in forthcoming research pursuits. We intend to investigate more extended operators, including dynamic Dombi aggregation operators and dynamic ordered weighted averaging/geometric operators. Furthermore, we aim to examine the effects of dynamic operators on advanced structures like intuitionistic fuzzy rough, q-rung fuzzy, picture and spherical fuzzy environment.

### Human and animal rights

The authors confirm that their study does not involve any humans/study participants or subjects/patients.

## Data Availability

All data generated or analyzed during this study are included in this article.
